# Population genetics analysis of Tolai hares (*Lepus tolai*) in Xinjiang, China using genome-wide SNPs from SLAF-seq and mitochondrial markers

**DOI:** 10.3389/fgene.2022.1018632

**Published:** 2023-03-20

**Authors:** Miregul Mamat, Wenjuan Shan, Pengcheng Dong, Shiyu Zhou, Peng Liu, Yang Meng, Wenyue Nie, Peichen Teng, Yucong Zhang

**Affiliations:** Xinjiang Key Laboratory of Biological Resources and Genetic Engineering, College of Life Science and Technology, Xinjiang University, Urumqi, China

**Keywords:** *Lepus tolai*, SLAF-seq, mtDNA, genetic diversity, genetic structure

## Abstract

The main topic of population genetics and evolutionary biology is the influence of the ecological environment, geographical isolation, and climatic factors on population structure and history. Here, we estimated the genetic diversity, genetic structure, and population history of two subspecies of Tolai hares (*Lepus tolai* Pallas, 1778), *L. t. lehmanni* inhabiting Northern and Northwest Xinjiang and *L. t. centrasiaticus* inhabiting Central and Eastern Xinjiang using SNP of specific-length amplified fragment sequencing (SLAF-seq) and four mitochondrial DNA (mtDNA). Our results showed a relatively high degree of genetic diversity for Tolai hares, and the diversity of *L. t. lehmanni* was slightly higher than that of *L. t. centrasiaticus*, likely due to the more favorable ecological environment, such as woodlands and plains*.* Phylogenetic analysis from SNP and mtDNA indicated a rough phylogeographical distribution pattern among Tolai hares. Strong differentiation was found between the two subspecies and the two geographical groups in *L. t. centrasiaticus*, possibly due to the geographical isolation of mountains, basins, and deserts. However, gene flow was also detected between the two subspecies, which might be attributed to the Tianshan Corridor and the strong migration ability of hares. Tolai hare population differentiation occurred at approximately 1.2377 MYA. Population history analysis based on SNP and mtDNA showed that the Tolai hare population has a complex history and *L. t. lehmanni* was less affected by the glacial event, possibly because its geographic location and terrain conditions weaken the drastic climate fluctuations. In conclusion, our results indicated that the joint effect of ecological environment, geographic events, and climatic factors might play important roles in the evolutionary process of *L. t. lehmanni* and *L. t. centrasiaticus*, thus resulting in differentiation, gene exchange, and different population history.

## Introduction

Climate factors and habitat environment will profoundly impact the evolution of biological populations, thus leaving historical traces in the genetic diversity, population structure, distribution pattern, and other aspects of today’s populations. For example, climate changes profoundly affected phylogeographic structure and the evolutionary history of brown hares (*L. europaeus*) by isolating populations in the distinct refugia where they adapted and differentiated in allopatry, leading to genome incompatibilities (Djan et al., 2017; Minoudi et al., 2018; Giannoulis et al., 2019). The southwest of Tarim Basin in Xinjiang, China, as the origin of rivers in the basin, was a glacial refugia for Yarkand hares (*L. yarkandensis*) during the Quaternary climate oscillations, providing a suitable environment for maintaining the relatively high genetic diversity of this species (Shan et al., 2011). In addition, genome-wide SNPs have confirmed differentiation between the southwest and northern populations (Ababaikeri et al., 2021) and higher diversity in the former population.

*Lepus* species have a wide distribution and can survive in various complex terrestrial habitats (Ben Slimen et al., 2018). Hence, they are indispensable for local ecosystems as part of the food chain. The Tolai hare (*Lepus tolai* Pallas, 1778) has sandy yellow, brownish gray, or gray dorsal pelage with a dark ripple (Smith et al., 2018). It occupies in various habitats, including desert, semi-desert, mountain steppe, forest-steppe, rocky habitats, and grasslands, and ranges from low to high elevations. Their strong ability to survive in diverse habitats makes them a good model for studying animal adaptation to the environment. However, since the low differences in morphology, and hybridization among hares, their classification has been challenging (Liu et al., 2011). Tolai hares were once classified as Cape hares (*L. capensis*) or Brown hares, but studies based on molecular biology (Wu et al., 2005; Wang and Yang, 2012; Shan et al., 2020b) and skull measurements (Cheng et al., 2012) did not support the classification of “*L. capensis*” in China as *L. capensis*. Therefore, the original Xinjiang’s “*L. capensis*” population was divided into *L. tibetanus* and *L. tolai*, and the population distributed in the north of the Tianshan Mountains was classified as *L. tolai* (Shan et al., 2020a).

Xinjiang, China, is in the hinterland of the Eurasian continent, with a dry climate and unique geological structure called “three mountains nip two basins”. Tolai hare distributes in the vast area of northern, central, and northwest in Xinjiang (Smith and Xie, 2008; Smith et al., 2018) including Altai Mountains and Junggar Basin in the north, Turpan-Hami Basin in the east, and the Tianshan Mountains crossing the central-eastern part of Xinjiang. Recent studies have shown two subspecies of Tolai hare in Xinjiang, with only slight different appearances (Smith et al., 2018; Shan et al., 2020a), among which *L. t. lehmanni* mainly inhabits the northern and northwestern regions. Except for Junggar Basin, these areas have many rivers, abundant water resources, and relatively humid climates. *L. t. centrasiaticus* mainly distributes in the central and eastern Tianshan Mountains. These areas are relatively dry with little rainfall and are dominated by arid and desert habitats.

Early molecular biological studies of Tolai hares were based on the misclassification of *L. tolai* into *L. capensis*. For example, Wu et al. (Wu et al., 2005) studied the phylogenetic relationships, biogeographic distribution, and species origin patterns between Chinese hare groups, including “*L. capensis*” in central Xinjiang. Wang et al. (Wang and Yang, 2012) sequenced the entire mitochondrial genome of Cape hares. They reconstructed phylogenetic relationships in genus *Lepus* based on *CYTB*, including so-called *“L. capensis”* distributed in Xinjiang. Liu et al. (Liu et al., 2011) used four mitochondrial DNA (mtDNA) fragments and nuclear gene to demonstrate that frequent introgression occurred through historical and recent interspecific hybridization among six Chinese hare species, including those in northern Xinjiang. Wu et al. (Wu et al., 2011) found extensive bidirectional mitochondrial DNA and SRY gene introgression in hybrids of Yarkand hare and Xinjiang “*L. capensis*”. Recently, some studies have mapped the full mitochondrial genome of the Xinjiang Tolai hare (Shan et al., 2020b). However, there has been no comprehensive evaluation of their genetic diversity and structure related to habitat and climate changes, and research on the biology of this species is scarce.

Specific-Locus Amplified Fragment sequencing (SLAF-seq) is a high throughput, high-resolution, and low-cost marker development technology that has emerged recently (Sun et al., 2013). This technique focuses on finding single nucleotide polymorphisms (SNPs), an abundant form of genetic variation, in an economical way (Zhang and Zhang, 2005; Wang et al., 2016; Ali et al., 2018; Qin et al., 2019). SNPs are found throughout the genome, and their distribution can reflect the population’s genetic variation. SLAF-seq has been used to analyze several species’ genetic diversity and phylogenetic structure (Li et al., 2017; Chang et al., 2019; Qin et al., 2019; Zhang J. et al., 2020a; Fang et al., 2020; Ababaikeri et al., 2021). In addition, mtDNA provides a different perspective of population genetic structure because it is maternally inherited and generally lacks intermolecular recombination (Allendorf, 2017).

This study used SLAF-seq to identify genome-wide SNP markers and combined them with four mtDNA markers (*COI*, *ND4*, *CYTB*, and D-LOOP) to evaluate the genetic diversity of two subspecies of Tolai hare in different habitats and reveal the effects of ecological environment, geographical isolation, and climate change on population structure characteristics and population demography history. This study will help to understand the evolutionary history of this species and provide essential data to maintain the biodiversity and stability of the Xinjiang ecosystem.

## Materials and methods

### Sampling and DNA extraction

**FIGURE 1:**
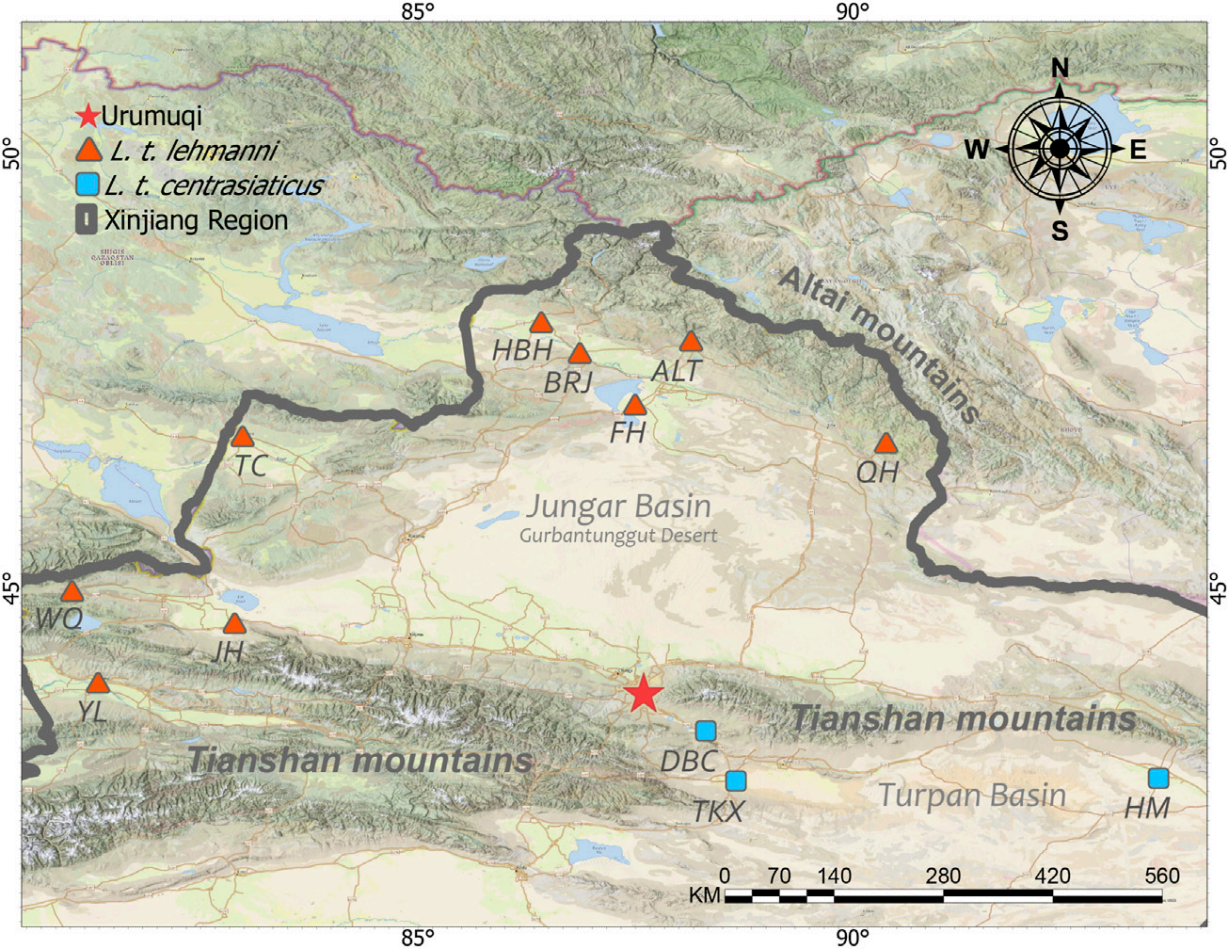
Approximate sampling sites of Tolai hare populations in Xinjiang, China.

Muscle or skin tissue samples were collected from 106 Tolai hares from 12 geographic populations in northern, northwestern, central, and eastern Xinjiang from 2008 to 2019 (Table 1 and Figure 1). The 81 *L. t. lehmanni* samples included 59 samples from Altay Prefecture (24 from Altay, 26 from Fuhai, three from Burqin, four from Habahe, and two from Qinghe), three from Tarbagatay Prefecture, 13 from Bortala Mongol Autonomous Prefecture (including 12 from Jinghe and one from Wenquan), and six from Ili. Twenty-five *L. t. centrasiaticus* samples included 11 from Dabancheng, two from Tuokexun, and 12 from Kumul. The samples were divided into northern, northwest, central, and eastern groups, and the geographical details of the sampled populations are shown in Table 1. All Tolai hare samples were used for mitochondrial DNA analysis. For SLAF-seq analysis, samples that were stored for a long time, severely degraded, or whose DNA quality was too low to be sequenced were eliminated, and a total of 36 Tolai hare samples in two subspecies remained. These included samples from Altay, Tarbagatay Prefecture, Ili, Dabancheng, and Tuokexun (Table 1). Some samples in this study were confiscated from poachers and provided to us by local forestry bureaus, while others came from hares that died of natural causes. All experimental protocols involved in this study were approved by the Institutional Animal Care and Use Committee of the College of Life Science and Technology, Xinjiang University, Urumqi, China.

**TABLE 1:** Description of analyzed Tolai hare samples from Xinjiang.

Subspecies	Geographical grouping	Sampling site	Samples for SLAF-seq	Samples for mtDNA
*L. t. lehmanni*	Northern group	Altay (ALT)	19	24
		Burqin (BRJ)	—	3
		Fuhai (FH)	—	26
		Habahe (HBH)	—	4
		Qinghe (QH)	—	2
	Northwest group	Tarbagatay (TC)	2	3
		Jinghe (JH) and Wenquan (WQ)	—	13
		Ili (YL)	2	6
*L. t. centrasiaticus*	The central group	Dabancheng (DBC)	11	11
		Tuokexun (TKX)	2	2
	Eastern group	Kumul (HM)	—	12
Total	4	12	36	106

Muscle samples were preserved in sterile tubes with anhydrous alcohol at -80°C until total genomic DNA extraction using a DNA tissue extraction kit. Genomic DNA integrity was determined using 1.0% agarose gel electrophoresis.

### Construction of SLAF-seq library and high-throughput sequencing

The domestic rabbit (*Oryctolagus cuniculus*) OryCun 2.0 genome (Lindblad-Toh et al., 2011) from the National Center for Biotechnology Information (NCBI: ftp://ftp.ncbi.nlm.nih.gov/genomes/all/GCF/000/003/625/GCF_000003625.3_OryCun2.0/) served as the reference genome for genome electronic enzyme digestion prediction. The selection principle for the enzyme digestion scheme was as follows: the proportion of restriction fragments located in the repetitive sequence was as low as possible, the restriction fragments were distributed as evenly as possible across the genome, the length of restriction fragments was highly consistent with the experimental study system, and the number of restriction fragments obtained matched the expected number of tags. A RsaI-EcoRV-HF^®^ restriction enzyme was used to digest the genomic DNA. From the constructed SLAF library (Sun et al., 2013; Zhang et al., 2013) DNA fragments 314–344 base pairs (bp) long were selected for sequencing on an Illumina HiSeq 2,500 system (Illumina, Inc., San Diego, CA, United States) at Beijing Biomarker Technologies Corporation (Beijing, China). The *Oryzasativa ssp. japonica* genome (http://rapdb.dna.affrc.go.jp/) was selected as the positive control for sequencing, and SOAP v2 software (Li R. et al., 2009b) was used to calculate the enzyme digestion efficiency, fragment insertion distribution, and alignment efficiency to evaluate the reliability of the enzyme digestion experiment and the accuracy of library construction.

### Quality control and SNP calling

Reads were obtained for each sample, and after filtering the connectors of the sequencing reads, the sequencing and data volumes were evaluated. We calculated the ratio of high-quality reads based on two key quality indicators, Q30 (read quality score of 30, indicating a base sequencing error probability of 0.1%) and GC content. All sample reads were mapped to the OryCun 2.0 genome sequence using BWA v0.7.5a-r405 (Li and Durbin, 2010). Then, we mined the SLAF tags according to the restriction fragment size defined by the enzyme digestion scheme. SNP calling was performed using GATK v3.3.2 (McKenna et al., 2010) and SAM tools v0.1.18 (Li H. et al., 2009a), and the intersection of SNP markers called by the two packages was chosen to build the original SNP dataset. Plink v1.07 (Purcell et al., 2007) was used to filter high-quality SNPs according to the criteria of site information integrity (INT) ≥ 0.5 and minor allele frequency (MAF) ≥ 0.05. Finally, the selected high-quality SNPs were used for further analysis.

### Mitochondrial DNA sequencing

The *COI*, *ND4*, *CYTB*, and D-LOOP fragments of mitochondrial DNA were amplified. *COI* PCR primers were 5′-AGGAACAGCCCTYAGTCT-3′ (Forward, F) and 5′-GGT​GGG​CTC​AAA​CAA​TAA-3′ (Reverse, R) (Zhang Y. et al., 2020b). PCR primers for *ND4* were 5′-GCAAAGAAT​CAT​TACTACGCAAA-3′ (F) and 5′-TTGCGACGATT​ACTAAGG​CTA-3′ (R) (Zhang Y. et al., 2020b). PCR primers for *CYTB* were 5′-CGA​ACC​CCA​CAA​AAC​CAA​TTA​C-3′ (F) and 5′-GGT​GAG​TTG​ATC​TCC​GTT​TCT​G-3′ (R), *CYTB* primers were designed by ourselves based on the published mitochondrial genome of Tolai hare. PCR primers for the D-LOOP were 5′-CAGAGATGGAGATYAACTC-3′ (F) and 5′-GCA​TGG​GCT​GAT​TAG​TCA​T-3′ (R) (Shan et al., 2011). The PCR amplification reaction comprised 13 µL Premix Taq (1.25U · 25 μL-1), 1 µL each forward and reverse primers (10 μmol·L-1), 1 µL DNA template, and 9 µL sterile deionized water. PCR amplification included denaturation at 95°C for 3–5 min, followed by 25–35 cycles of denaturation at 94°C for 30 s, annealing at 51°C for 30 s, and extension at 72°C for 1 min. A final extension was made at 72°C for 10 min. The PCR products were identified using 1.5% agarose gel electrophoresis, and PCR products with good amplification results were sequenced. The mtDNA gene sequences were aligned by MAFFT and combined using Phylosuite v1.2.2 (Zhang et al., 2018).

### Data analysis

Summary statistics describing genetic diversity, including nucleotide diversity (*π*), observed heterozygosity (*Ho*), excepted heterozygosity (*He*), and the polymorphism information content (*PIC*) were calculated using Power-marker v3.25 (Liu and Muse, 2005) and Arlequin ver3.5 (Excoffier and Lischer, 2010).

Phylogenetic analysis based on SNPs was reconstructed using the Neighbor-Joining (NJ) method and Maximum-Likelihood (ML) method. The NJ phylogenetic tree was performed in Mega X (Kumar et al., 2018) with 1,000 bootstraps, and the ML phylogenetic tree was performed in IQ-TREE (Nguyen et al., 2015) with 3,000 bootstraps on the Cipres (https://www.phylo.org/index.php/). mtDNA-based phylogenetic analysis was conducted on the Cipres using the Bayesian Inference (BI) method by MrBayes 3.2.7a (Ronquist et al., 2012) with five million Bayesian Markov chain Monte Carlo (MCMC) generations and ML tree by RAxML-NG (Kozlov et al., 2019) with 1,000 bootstraps. The rabbit (*O. cuniculus*) genome was used as the outgroup (GenBank accession: GCF_000003625.3 for SNP and AJ001588.1 for mtDNA). ModelFinder was used to identify the best fit base-pair substitution model according to the Bayesian information criterion (BIC) using Phylosuite v1.2.2 (Zhang et al., 2018), and the general time-reversible (GTR) model was selected as the optimal model for phylogenetic analysis of the SNPs and mtDNA concatenated dataset. Principal component analysis (PCA) was conducted using Plink v1.07 (Purcell et al., 2007). The population structure among the Tolai hare populations was inferred using ADMIXTURE v1.3.0 (Alexander et al., 2009). Treemix v.1.13 (Pickrell and Pritchard, 2012) was used to infer multiple population splitting and mixing events using genome-wide allele frequency data. The median-joining network was conducted by Popart (Leigh and Bryant, 2015). We performed a hierarchical analysis of molecular variation (AMOVA) using Arlequin ver3.5 (Excoffier and Lischer, 2010).

Divergence times were estimated based on mtDNA using Beast v1.10.4 (Suchard et al., 2018). Four points were calibrated to build the time tree: the split of *Lepus* (approximately 11.57 million years ago, MYA), the divergence time of *L. americanus* (approximately 8.6 MYA), the divergence time of *L. europaeus* (approximately 1.84 MYA) (Ge et al., 2013), and Tolai hare fossil calibration (0.78 MYA) (Erbajeva and Alexeeva, 2000). Best substitution models according to BIC (HKY model for *COI*, *ND4*, and *CYTB* and GTR model for D-LOOP) were found using modelFinder in Phylosuite v1.2.2 (Zhang et al., 2018), and uncorrelated relaxed lognormal clock with a prior coalescent tree of constant size was used. The Markov chain Monte Carlo analysis was run thrice for 1 × 10^8^ generations, sampling every 1,000 generations. Tracer v1.7.2 (Rambaut et al., 2018) was used to check the log files and ensure that the effective sample size (ESS) for all parameters exceeded 200. TreeAnnotator.v1.10.4 (Suchard et al., 2018) was used to summarize the tree data, and the first 10% of trees were discarded as a burn-in. The tree and divergence times were displayed and edited in Figtree v1.4.3 (http://tree.bio.ed.ac.uk/software/figtree/). SMC++ v1.15.5 (Terhorst et al., 2017) was used to check the population history of the two subspecies based on SNPs. Tajima’s D and Fu’s *Fs* analyses were performed to test neutrality based on mtDNA in Arlequin ver3.5 (Excoffier and Lischer, 2010). The mismatch distributions of pairwise sequence differences for the Tolai hare populations were estimated using DnaSP6 (Rozas et al., 2017). To estimate changes in effective population size through evolutionary time, we explored population history by constructing Extended Bayesian Skyline Plots (EBSP) in Beast. v2.4.7 (Bouckaert et al., 2014) with mtDNA. The prior tree was set as the Coalescent Extended Bayesian Skyline and sampled every 1,000 steps for 1 × 10^8^ steps. The ESS values for all the parameters were assessed using Tracer v1.7.2 (Rambaut et al., 2018).

## Results

### SLAF-seq and SNP discovery

The rabbit genome served as the reference genome for predicting electron enzymes and identified a restriction fragment length of 314–344 bp, ultimately defined as a SLAF tag. Sequencing results for the positive control indicated that the efficiency of paired-end comparison was 96.84%, and the enzyme digestion efficiency of the control was 94.74%, indicating that the process was normal and reliable.

High-throughput sequencing of the SLAF library yielded 226.85 Mb of high-quality clean data after strict filtration (Additional file 1: Supplementary Table S1). The average Q30 was 95.39%, indicating that the results of the tested sequences were reliable (Additional file 1: Supplementary Table S1). The average GC content was 41.80%, and the average mapping rate of our samples to the reference genome was 96.13% (Additional file 1: Supplementary Table S1).

A total of 2,205,716 SLAF tags were obtained, with an average sequencing depth of 16.93× (Additional file 1: Supplementary Table S1). A total of 2,005,461 SNPs were obtained from 36 samples, and SNP integrity ranged from 31.59% to 49.50%, averaging 42.72%. SNP heterozygosity ranged from 4.48% to 8.22%, with an average of 5.61% (Additional file 1: Supplementary Table S1). To reduce sequencing errors, eliminate baseline differentiation, and evaluate the accuracy, 473,241 consistent and high-confidence SNPs were selected for further analysis according to INT ≥0.5 and MAF ≥0.05. Accession number of SNP data for the Xinjiang Tolai hare provided in Additional File 2: Supplementary Table S2.

### Genetic diversity and population structure analysis of SLAF-seq

Nucleotide diversity (*π*) ranged from 0.0444 (TC population) to 0.0823 (YL population) across the five geographic populations of Tolai hare in Xinjiang and averaged 0.05926 per population (Table 2). The average *He*, *Ho*, and *PIC* values of all populations were 0.37412, 0.40226, and 0.2974, respectively (Table 2). The northwest group had the highest genetic diversity (*π* = 0.06335, *He* = 0.42115, *Ho* = 0.48865, *PIC* = 0.33065). In general, the genetic diversity indices of *L. t. lehmanni* (*π* = 0.0619, *He* = 0.3811, *Ho* = 0.4061, *PIC* = 0.3019) were slightly higher than those of *L. t. centrasiaticus* (*π* = 0.0553, *He* = 0.3637, *Ho* = 0.3965, *PIC* = 0.2906) (Table 2).

**TABLE 2:** Summary statistics describing Xinjiang’s Tolai hare genetic diversity based on SNPs.

Subspecies	Geographical grouping	Population (abbreviation)	Nucleotide diversity (*π*)	Expected heterozygosity (*He*)	Observed heterozygosity (*Ho*)	Polymorphism information content (*PIC*)
*L. t. lehmanni*	Northern	ALT	0.0590	0.3009	0.2410	0.2445
	Northwest	TC	0.0444	0.4203	0.5314	0.3302
		YL	0.0823	0.4220	0.4459	0.3311
		Mean	0.06335	0.42115	0.48865	0.33065
*L. t. centrasiaticus*	Central	DBC	0.0609	0.3166	0.2690	0.2564
		TKX	0.0497	0.4108	0.5240	0.3248
		Mean	0.0553	0.3637	0.3965	0.2906
Total	—	Mean	0.05926	0.37412	0.40226	0.2974

**FIGURE 2:**
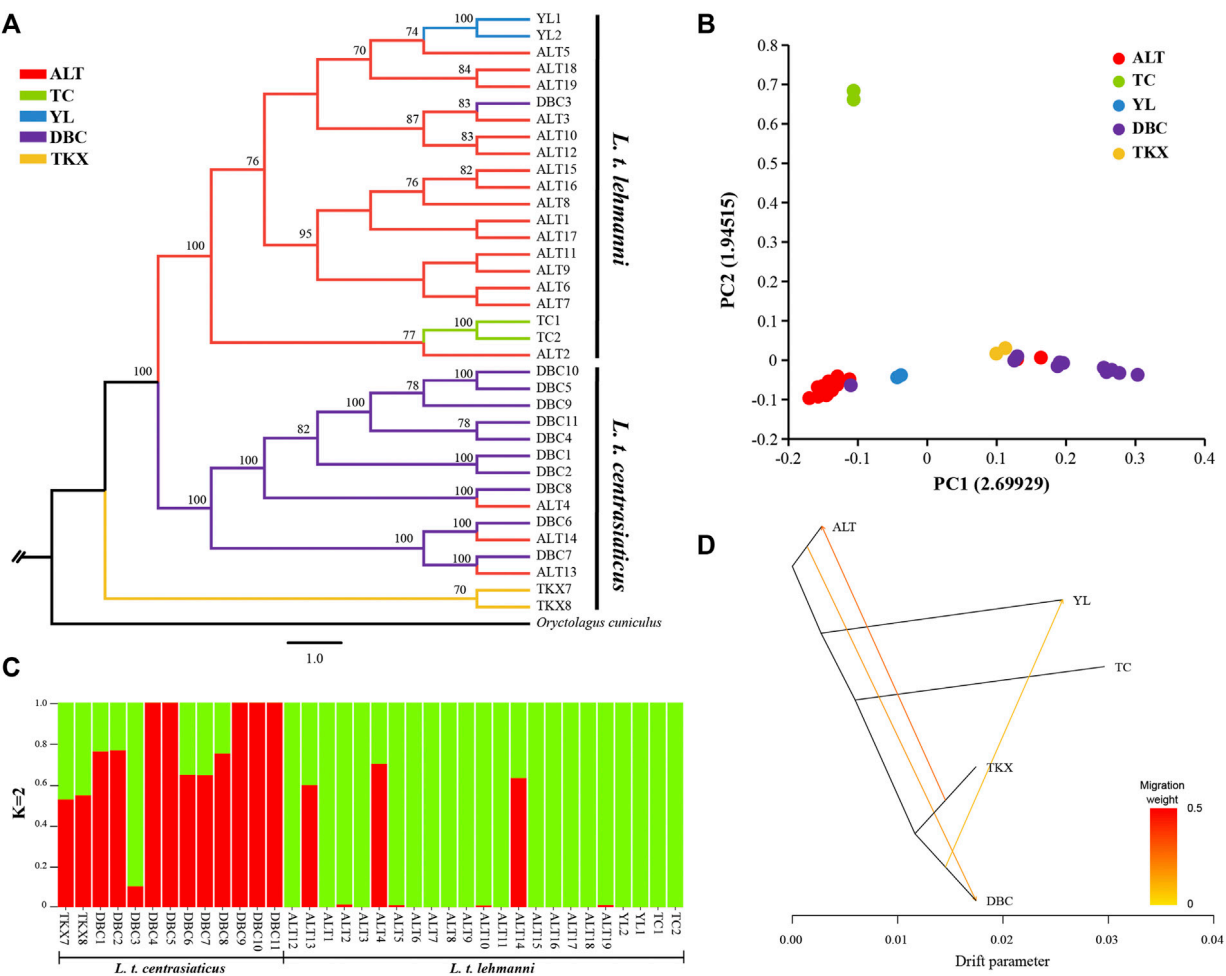
Phylogenetic analysis and population structure of Tolai hares in Xinjiang based on SNPs. **(A)**. Phylogenetic tree constructed using the ML method. **(B)**. Principal component analysis (PCA). **(C)**. Genetic Structure of Tolai hares. The number of ancestral populations (K=2) is indicated. **(D)**. Inferred Tolai hare phylogenetic tree with mixture events among populations. Arrows indicate migration events and are colored according to their migration weight.

We reconstructed phylogeny to explore genome-wide relationships among Tolai hare populations. The topology of ML (Figure 2A) and NJ trees (Additional file 3: Supplementary Figure S1) was consistent. The phylogenetic tree results showed that the Tolai hares analyzed in this study based on SNPs were divided into two main clusters with high confidence, including three clades. The first branch was located at the tree’s root, containing two samples from the TKX population. The second branch consisted of samples from the DBC population and three individuals from the ALT population. These two clades were grouped into one cluster because it included most samples from the central group of *L. t. centrasiaticus*, except for three individuals from the ALT population of *L. t. lehmanni*. The third branch consisted mainly of samples from the northern and northwestern groups, including samples from the ALT, TC, and YL populations of *L. t. lehmanni*, and one sample from the DBC population. The clustering relationships among populations were also evident in the PCA, in which *L. t. centrasiaticus* individuals were clustered, and *L. t. lehmanni* individuals were clustered together except for individuals from the TC population (Figure 2B). The population relationships were largely consistent with the geographical distribution of the samples, consistent with the phylogenetic tree.

To evaluate population structure, the ADMIXTURE was performed (Figure 2C, Additional file 4: Supplementary Figure S2). Since the cross-validation errors at K = 2 is close to the lowest cross-validation errors at K = 1 (Additional file 5: Supplementary Figure S3), the assessment of the population structure indicated a clear subdivision of two main ancestral lineages when K = 2. Except for DBC3, most samples in *L. t. lehmanni* formed an ancestral cluster (shown in green in Figure). Most samples in *L. t. centrasiaticus*, and three ALT individuals formed another ancestral cluster (shown in red in the image). In addition, fifteen samples have mixed lineages.

Genetic differences among the geographical groups were examined using AMOVA (Table 3), and the results indicated moderate genetic differences among the geographical groups (7.65%, *F*_ST_ = 0.0765, *p* < 0.01). When individuals were pooled into the northern group and northwest group, only 6.57% (*p* < 0.01) of the variability was partitioned among groups, and differentiation among groups was 0.0657 (*p* < 0.01). When individuals were pooled into the *L. t. lehmanni* and *L. t. centrasiaticus*, only 6.57% (*p* < 0.01) of the variability was partitioned among populations, and differentiation among subspecies was 0.0657 (*p* < 0.01).

**TABLE 3:** AMOVA of the Tolai hare groups in Xinjiang based on SNPs.

Groups	*F*_ST_	Percentage of variation (%)	
		Among groups	Within group
[Northern] [Northwest] [Central]	0.0765**	7.65**	92.35
[Northern] [Northwest]	0.0657**	6.57**	93.43
[*L. t. lehmanni*] [*L. t. centrasiaticus*]	0.0657**	6.57**	93.43

*p < 0.05 **p < 0.01.

To further infer population gene flow, we performed a Treemix analysis based on SNPs (Figure 2D). Three migrations were detected. One event began from the TKX to the ALT population (migration weight 0.2775). The second began from the ALT population to the DBC population (migration weight 0.184,875). The third event is from the DBC to the YL population (migration weight 0.0999,906).

### Genetic diversity and population structure of mtDNA

**FIGURE 3:**
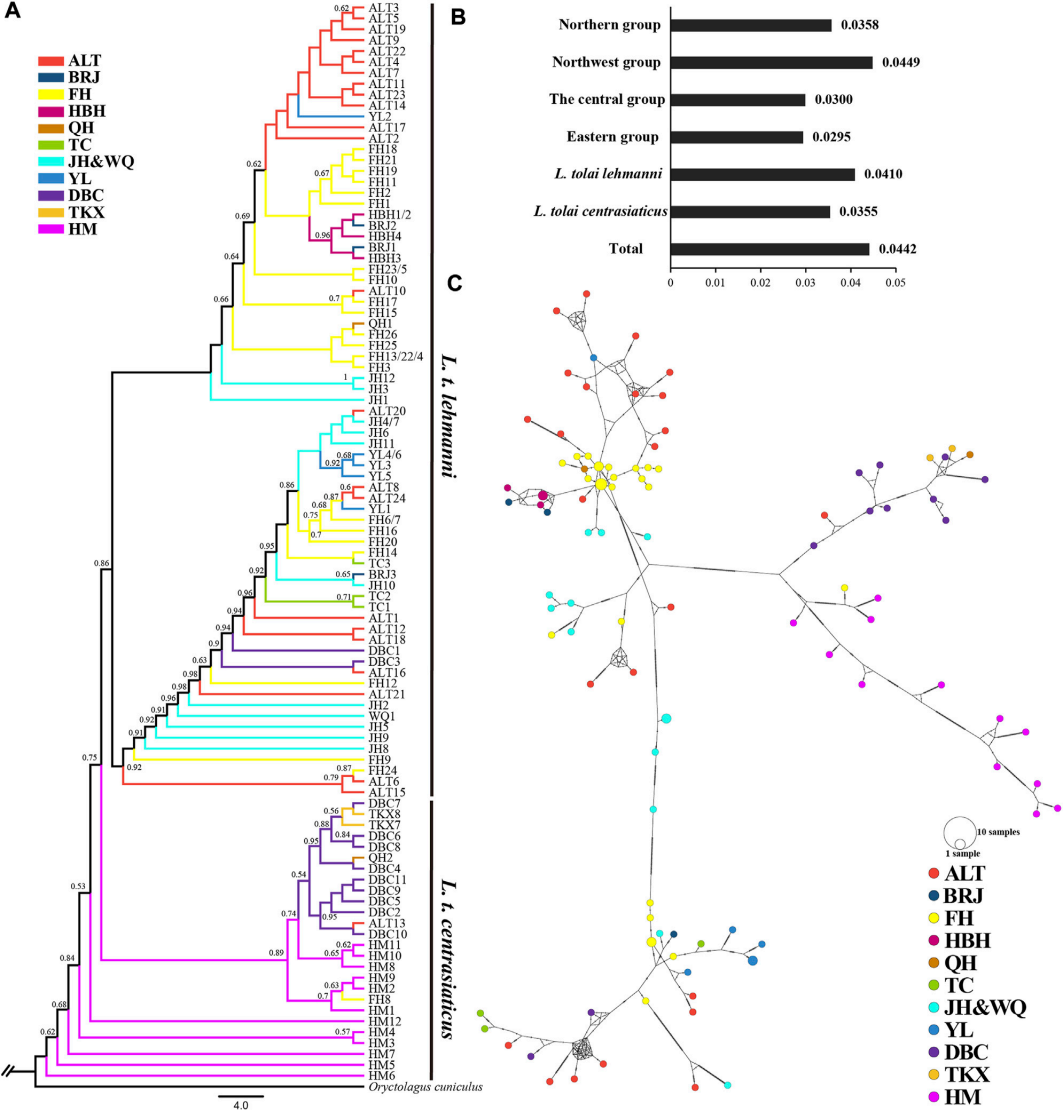
Genetic diversity and population structure of Tolai hares in Xinjiang based on mtDNA. **(A)**. Phylogenctic tree constructed using ML method. **(B)**. Nucleotide diversity (π). **(C)**. Median-joining network of Tolai hare haplotypes. The hatch marks on the line indicates the mutation numbers.

Sequencing of the four mitochondrial DNA segments (*COI*, *ND4*, *CYTB*, and D-LOOP) with an alignment length of 2,885 bp in 106 Tolai hare individuals resulted in 99 haplotypes. Nucleotide diversity ranged from 0.0295 (eastern group) to 0.0449 (northwest group) (Figure 3B). The *π* value of *L. t. lehmanni* (0.0410) was slightly higher than that of *L. t. centrasiaticus* (0.0355) (Figure 3B). The total *π* value of the Tolai hare in Xinjiang was 0.0442.

To explore the phylogenetic structure, ML (Figure 3A) and BI evolutionary trees (Additional file 6: Supplementary Figure S4) were constructed, and the topological structures of the two trees were consistent. The phylogenetic tree based on combined haplotype sequences of four mitochondrial genes showed that the Tolai hare samples analyzed in this study contained four branches and could be further divided into two main clusters (Figure 3A). The first cluster was located at the tree’s root and predominantly comprised of *L. t. centrasiaticus* individuals from the central and eastern groups. Each individual from the QH, ALT, and FH populations were included. The second cluster was comprised of *L. t. lehmanni* individuals from the northern and northwest groups, and two individuals from the DBC population. In addition to the evolutionary tree, a median-joining network was constructed to further elucidate the phylogenetic relationships among haplotypes (Figure 3C). The haplotype grouping in the median-joining network was consistent with the clustering in the evolutionary tree. The network verified the existence of two distinct clades separated by several mutational steps.

Genetic differentiation among the groups was next examined using AMOVA. The results showed that when pooling individuals into northern, northwest, central, and eastern groups, the genetic variation among groups was 26.00% (*p* < 0.01), and differentiation was 0.2600 (*p* < 0.01) (Table 4). Genetic variation and differentiation among groups was lower when individuals were pooled into the northern and northwest group (15.58%, *F*_ST_ = 0.1558, *p* < 0.01) than when individuals were pooled into the central and eastern group (27.24%, *F*_ST_ = 0.2724, *p* < 0.01) (Table 4). In addition, when individuals pooled into *L. t. lehmanni* and *L. t. centrasiaticus*, genetic variation among populations was 23.76% (*p* < 0.01), and *F*_ST_ was 0.2376 (*p* < 0.01).

**TABLE 4:** AMOVA of Tolai hare groups in Xinjiang based on mtDNA.

Groups	*F*_ST_	Percentage of variation (%)	
		Among groups	Within group
[Northern] [Northwest] [Central] [Eastern]	0.2600**	26.00**	74.00
[Northern] [Northwest]	0.1558**	15.58**	84.42
[Central] [Eastern]	0.2724**	27.24**	72.76
[*L. t. lehmanni*] [*L. t. centrasiaticus*]	0.2376**	23.76**	76.24

*p < 0.05; **p < 0.01.

### Divergence time estimation and population history

**FIGURE 4:**
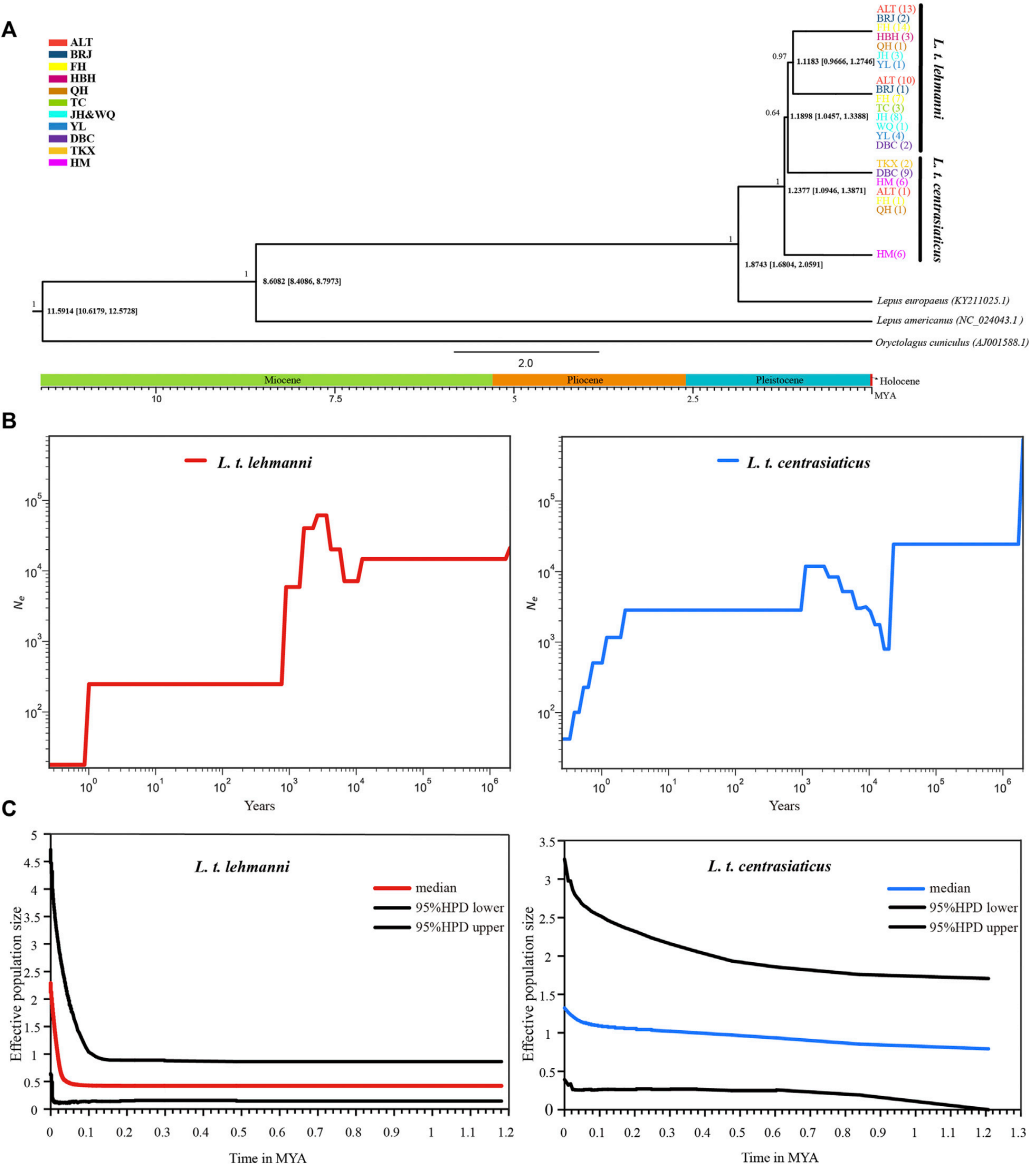
Divergence time and population history of Tolai hare populations. **(A)**. Time scale of Tolai hare populations divergence in Xinjiang based on mtDNA. Posterior probabilities are shown above nodes, divergence time with 95% highest posterior densities (HPD) are shown below nodes. **(B)**. SMC++ for *L. t. lehmanni* and *L. t. centrasiaticus*. **(C)**. Historical demographic trends represented by Extended Bayesian Skyline Plot (EBSP) for *L. t. lehmanni* and *L. t. centrasiaticus*.

We used mtDNA data to construct a Tolai hare differentiation time-merging tree. The tree showed that Tolai hare population differentiation occurred at approximately 1.2377 MYA, and the eastern HM population showed the earliest differentiation. The divergence between *L. t. lehmanni* and *L. t. centrasiaticus* occurred at approximately 1.1898 MYA (Figure 4A).

To estimate the historical population dynamics of Tolai hare in Xinjiang, we performed SMC++ analysis to track changes in effective population sizes (*Ne*) over time based on SNPs. The dynamics of historic *Ne* for *L. t. lehmanni* and *L. t. centrasiaticus* are shown in Figure 4B. The effective population size of *L. t. centrasiaticus* decreased in the last glacial maximum (LGM), while *L. t. lehmanni* population remained relatively stable. Both subspecies expanded during the interglacial period after the last glacial period (LGP), especially *L. t. lehmanni*. About 0.001–0.0015 MYA, however, populations of both subspecies declined. In addition, the Tajima’s D, Fu’s *Fs*, mismatch distribution, and EBSP based on mtDNA were used to analyze the population history of Tolai hares. The neutrality test results showed that Tajima’s D and Fu’s *Fs* values for *L. t. lehmanni* were 2.06761 (*p* = 0.9800 > 0.05) and -7.73931 (*p* = 0.0480 < 0.05), and -0.00167 (*p* = 0.5720 > 0.05) and -3.95338 (*p* = 0.0310 < 0.05) for *L. t. centrasiaticus.* The mismatch distribution analysis revealed that *L. t. lehmanni* and *L. t. centrasiaticus* showed multi-peak curves (Additional file 7: Supplementary Figure S5). The demographic scenario for Tolai hare populations determined through EBSP analysis suggested a more pronounced population expansion of *L. t. lehmanni* beginning from 0.02 MYA, and the *L. t. centrasiaticus* population showed a less distinct tendency to expand (Figure 4C).

## Discussion

### The relatively high genetic diversity of Tolai hare populations

The amount of genetic diversity reflects the evolutionary potential of a species, and populations with more genetic diversity are expected to adapt better to environmental changes such as climate change, habitat loss, over-harvesting, invasive species, and disease than populations with low genetic diversity (Kardos, 2021). In this study, we used a set of SLAF-seq genome-wide SNP and mtDNA markers to estimate the genetic diversity of Tolai hares in Xinjiang. At the genome-wide level, the genetic diversity of Tolai hares based on SNPs was higher (Table 2) (*π* = 0.05926, *He* = 0.37412, *Ho* = 0.40226, *PIC* = 0.2974) than that of other reported species, including rabbits (*PIC* = 0.2–0.2281, *He* = 0.2511–0.2857, *Ho* = 0.3072–0.3418, (Ren et al., 2019), and Yarkand hares (*π* = 0.0655, *He* = 0.3130, *Ho* = 0.2852, *PIC* = 0.2543 (Ababaikeri et al., 2021). The estimated nucleotide diversity of Tolai hares (*π* = 0.0442) based on mtDNA was also relatively high compared to other *Lepus* taxa, such as brown hares (*π*_D-LOOP_ = 0.030, (Minoudi et al., 2018), Yarkand hares (*π*_D-LOOP_ = 0.033, *π*_*CYTB*_ = 0.008, (Shan et al., 2011), and Italian hares (*L. corsicanus*, *π*_D-LOOP_ = 0.018, (Pierpaoli et al., 2003). These higher genetic diversity values indicated that Tolai hare populations evolved under long-term favorable environmental conditions and remained relatively stable and genetically variable (Pironon et al., 2017). Larger effective population sizes may have also contributed to this (Kimura, 1983; Hague and Routman, 2016).

SNPs and mtDNA-based genetic diversity analysis of the subspecies showed that *L. t. lehmanni* polymorphism level was higher than that of *L. t. centrasiaticus*, likely due to a more favorable living environment and distribution (Table 2 and Figure 3B). In Xinjiang, *L. t. lehmanni* is mainly distributed in the northern and northwestern regions, which are more humid and include vast forests, grasslands, and croplands, providing more water and food for survival. While *L. t. centrasiaticus* is mainly distributed in the central and eastern Xinjiang, including the Turpan-Hami basin, which is primarily the Gobi desert; thus, survival there is likely more challenging.

### The coexistence of genetic differentiation and gene flow in Tolai hare populations

Geological formation events can cause physical barriers to disperse species, such as mountains and rivers, leading to divergence and speciation (Chaves et al., 2011). Thus, geographical isolation is an important factor in genetic differentiation (Duncan et al., 2015; Gao et al., 2021). The effects of geographic isolation on genetic structure have been reported in several species, such as *Sarcophanops* (Campillo et al., 2020), *Orestias ascotanensis* (Cruz-Jofré et al., 2016), *Weigela coraeensis* (Yamada and Maki, 2012), *L. capensis,* and *L. europaeus* (Ben Slimen et al., 2008)*.* In this study, phylogenetic trees based on SNP and mtDNA markers (Figures 2A, 3A) showed that Tolai hares were divided into two main clusters (*L. t. lehmanni* and *L. t. centrasiaticus*). Most samples from the same or adjacent regions clustered together, and their clustering relationships were consistent with their geographic distribution, indicating a rough phylogeographic distribution pattern. This was also supported by the network analysis (Figure 3C) and the PCA (Figure 2B). In addition, combined with the *F*_ST_ and significant *p*-values in AMOVA based on SNPs and mtDNA (Tables 3 and 4), differentiation occurred between subspecies *L. t. lehmanni* and *L. t. centrasiaticus*. These two subspecies distribution areas span across the Tianshan Mountains in Xinjiang. We speculate that the high mountains, the barren basins and deserts, and long geographical distances serve as geographical barriers that limit the dispersal and communication between two subspecies, leading to genetic divergence. In addition, the different populations may have evolved different ecotypes with different feeding habits or habitats, hindering gene exchange and leading to differentiation (Sarabia et al., 2021).

A strong differentiation was also found between the Central and Eastern groups in *L. t. centrasiaticus* according to the phylogenetic analysis and AMOVA results from mtDNA (Table 4). Although the Dabancheng (DBC) and Tuokexun (TKX) of the Central Group are adjacent to the Kumul (HM) of the Eastern Group, they are separated by the huge Turpan-Hami Basin and the Gobi Desert and are relatively distant geographically. Moreover, the food shortage caused by drought, little rain, and hot habitat further prevent the migration, spread, and exchange of hares, thus promoting differentiation.

In this study, the genetic structure analysis showed that geographical isolation caused by geographical barriers, such as distance, mountains, basins, and deserts, as well as the ecological environment of the habitat, affected the genetic structure of hare populations. This also occurs in *Yarkand hare* in Xinjiang. The population genetics of Yarkand hares based on SNPs (Ababaikeri et al., 2021) and mtDNA (Shan et al., 2011) showed a systematic geographical distribution pattern and genetic differentiation between the north and southwest populations, which may have been caused by geographical isolation and different living environment. It is concluded that geographical isolation and complex habitats can affect hare populations’ genetic structure and differentiation.

On the other hand, parapatric and sympatric subspecies or populations are accessible to gene flow, thus affecting the genetic structure and evolutionary history. Despite clear population differentiation and significant variation among subspecies and geographical groups, our phylogenetic analyses, PCA plot, ADMIXTURE, and Treemix results revealed a certain degree of lineage admixture and gene flow between subspecies. This is likely due to the strong adaptability to environmental changes, large effective population sizes, and relatively strong migration capability, promoting gene exchange among populations (Ababaikeri et al., 2021). Moreover, since ancient times, the Tianshan Corridor in the Silk Road has connected the northern and southern Xinjiang (Chen, 2014). Although Tianshan Mountains lie across Xinjiang, the biological corridors may contribute to admixture. Gene flow also has an important impact on population differentiation. Generally speaking, differentiation occurs when there is limited gene flow between populations or strong natural selection is sufficient to overcome the dilution effect of gene flow between populations on population differentiation (Nosil, 2008; Li et al., 2014). Many studies reported gene flow coexisting in species or population differentiation (Nadachowska and Babik, 2009; Ababaikeri et al., 2021). In short, the geographical factors and the properties of hares might contribute to the coexistence of genetic differentiation and exchange in Tolai hares.

### The complex population history of Tolai hare populations

Climate-driven environmental changes during the Pleistocene influenced the evolution of many terrestrial species (Meiri et al., 2020). However, the influence of climate fluctuation on the Tolai hares population history has not been studied. In this study, neutrality tests, nucleotide mismatch distribution, EBSP analysis based on mtDNA, and SMC++ analysis based on SNP were used to explore the population history of Tolai hares in Xinjiang. The integrated results indicated that the Tolai hare population has a complex history. SMC++ shows that *L. t. centrasiaticus* effective population size decreased during the LGM while *L. t. lehmanni* population remained stable. *L. t. lehmanni* was less affected by LGM might because it is distributed between the mountains, which cushion drastic climate fluctuations (Yuan et al., 2019). In the interglacial period after the LGP, the population of both subspecies had a small increase with the growth of *L. t. lehmanni* being more evident, consistent with the EBSP analysis based on mtDNA. The population of the two subspecies decreased from about 0.001 to 0.0015 MYA. Previous studies have shown that early humans in Xinjiang expanded into the present region during this period (Tan et al., 2022), and that the early diet of humans in northwest China included wild animals (Ren et al., 2017). Meanwhile, previous researchers believed that *Lepus* could be influenced by human activities (Ge et al., 2013). Therefore, We hypothesize that human activity during this period led to population decline in both subspecies.

Unlike other species in Europe and eastern China, such as the Brown hare (Minoudi et al., 2018), Panda (*Ailuropoda melanoleuca*) (Zhao et al., 2013), and Lizards (*Shinisaurus crocodilurus*) (Xie et al., 2022), our comprehensive indexes of two markers showed that *L. t. lehmanni* population remained stable, even in the LGM (0.0265–0.019 MYA) (Clark et al., 2009; Liang et al., 2017). Other studies have shown that among the Tibetan Plateau species, population size did not decline in the LGM (Liang et al., 2017), implying that the geographical distribution of species and climate oscillation indeed play an important role in population history.

Tolai hare differentiated at 1.2377MYA, and the divergence between subspecies occurred between 1.1183 and 1.1898MYA, later than the formation time of the Tianshan Mountains and the Turpan-Hami Basin (Li et al., 2006). Therefore, mountains and basins act as geographical barriers for gene exchange between hare populations, resulting in apparent differentiation between the two subspecies and within *L. t. centrasiaticus*.

However, the limitations of the SLAF-seq and the mtDNA fragments used in population history analysis should also be considered. In the future, whole genome resequencing and complete mitogenome sequencing will be performed to obtain more reliable results on the Tolai hare population history.

## Conclusion

This paper used SNP and mitochondrial markers to explore the effects of geological formation and environmental and climate factors on the Tolai hare. We found that Tolai hares have a high genetic diversity due to their strong adaption and migration ability. In addition, geological events such as the formation of mountains, basins, and other geographical factors led to differentiation and gene flow between subspecies *L. t. lehmanni* and *L. t. centrasiaticus*. Thus, it is due to the different geographical and geological conditions resulting in a relatively steady climate, making *L. t. lehmanni* stable and less affected by LGM compared to *L. t. centrasiaticus*.

## Data Availability

The datasets presented in this study can be found in online repositories. The names of the repository/repositories and accession number(s) can be found below: https://www.ncbi.nlm.nih.gov/bioproject/PRJNA850843. The Accession number of mtDNA data for the Xinjiang Tolai hare in this study see the Additional file 2: Supplementary Table S2.

## References

[B1] Ababaikeri B. Zhang Y. Dai H. Shan W. (2021). Revealing the coexistence of differentiation and communication in an endemic hare, *Lepus yarkandensis* (Mammalia, Leporidae) using specific-length amplified fragment sequencing. Front. Zool. 18, 50. 10.1186/s12983-021-00432-x 34565397 PMC8474959

[B2] Alexander D. H. Novembre J. Lange K. (2009). Fast model-based estimation of ancestry in unrelated individuals. Genome Res. 19, 1655–1664. 10.1101/gr.094052.109 19648217 PMC2752134

[B3] Ali I. Teng Z. Bai Y. Yang Q. Hao Y. Hou J. (2018). A high density SLAF-SNP genetic map and QTL detection for fibre quality traits in Gossypium hirsutum. BMC Genomics 19, 879. 10.1186/s12864-018-5294-5 30522437 PMC6282304

[B4] Allendorf F. W. (2017). Genetics and the conservation of natural populations: Allozymes to genomes. Mol. Ecol. 26, 420–430. 10.1111/mec.13948 27933683

[B5] Ben Slimen H. Suchentrunk F. Stamatis C. Mamuris Z. Sert H. Alves P. C. (2008). Population genetics of cape and Brown hares (*Lepus capensis* and *L. europaeus*): A test of petter's hypothesis of conspecificity. Biochem. Syst. Ecol. 36, 22–39. 10.1016/j.bse.2007.06.014

[B6] Ben Slimen H. Awadi A. Tolesa Z. G. Knauer F. Alves P. C. Makni M. (2018). Positive selection on the mitochondrial ATP synthase 6 and the NADH dehydrogenase 2 genes across 22 hare species (genus *Lepus*). J. Zool. Syst. Evol. Res. 56, 428–443. 10.1111/jzs.12204

[B7] Bouckaert R. Heled J. Kuhnert D. Vaughan T. Wu C. H. Xie D. (2014). Beast 2: A software platform for bayesian evolutionary analysis. PLoS Comput. Biol. 10, e1003537. 10.1371/journal.pcbi.1003537 24722319 PMC3985171

[B8] Campillo L. C. Manthey J. D. Thomson R. C. Hosner P. A. Moyle R. G. (2020). Genomic differentiation in an endemic Philippine genus (Aves: *Sarcophanops*) owing to geographical isolation on recently disassociated islands. Biol. J. Linn. Soc. Lond. 131, 814–821. 10.1093/biolinnean/blaa143 34690487 PMC8528567

[B9] Chang Y. He P. Wang H. Li H. Wang S. Li L. (2019). Application of high-throughput sequencing to evaluate the genetic diversity among wild apple species indigenous to shandong, China, and introduced cultivars. Plant Mol. Biol. Rep. 37, 63–73. 10.1007/s11105-019-01138-5

[B10] Chaves J. A. Weir J. T. Smith T. B. (2011). Diversification in Adelomyia hummingbirds follows Andean uplift. Mol. Ecol. 20, 4564–4576. 10.1111/j.1365-294X.2011.05304.x 21981387

[B11] Chen T. (2014). “The Silk Road: The initial section and tianshan corridor network" highlights the study of universal values”. China Cult. Herit. (3), 72–81. (in Chinese)

[B12] Cheng C. Ge D. Xia L. Zhou C. Yang Q. (2012). Morphometrics study on the so called ‘cape hare’ (lagomorpha: Leporidae: *Lepus*) in China. Acta Theriol. Sin. 32, 275–286. 10.16829/j.slxb.2012.04.001 (in Chinese)

[B13] Clark P. U. Dyke A. S. Shakun J. D. Carlson A. E. Clark J. Wohlfarth B. (2009). The last glacial maximum. Science 325, 710–714. 10.1126/science.1172873 19661421

[B14] Cruz‐Jofré F. Morales P. Vila I. Esquer‐Garrigos Y. Hugueny B. Gaubert P. (2016). Geographical isolation and genetic differentiation: The case of *Orestias ascotanensis* (teleostei: Cyprinodontidae), an andean killifish inhabiting a highland salt pan. Biol. J. Linn. Soc. 117, 747–759. 10.1111/bij.12704

[B15] Djan M. Stefanovi M. Velikovi N. Lavadinovi V. Paulo C. A. Suchentrunk F. (2017). Brown hares (*Lepus europaeus* Pallas, 1778) from the balkans: A refined phylogeographic model. Hystrix-italian J. Mammal. 28, 186–193 10.4404/hystrix-28.2-12202

[B16] Duncan C. J. Worth J. R. P. Jordan G. J. Jones R. C. Vaillancourt R. E. (2015). Genetic differentiation in spite of high gene flow in the dominant rainforest tree of southeastern Australia, *Nothofagus cunninghamii* . Heredity 116, 99–106. 10.1038/hdy.2015.77 26350630 PMC4675879

[B17] Erbajeva M. A. Alexeeva N. V. (2000). Pliocene and Pleistocene biostratigraphic succession of Transbaikalia with emphasis on small mammals. Quat. Int. 68, 67–75. 10.1016/s1040-6182(00)00033-1

[B18] Excoffier L. Lischer H. E. (2010). Arlequin suite ver 3.5: A new series of programs to perform population genetics analyses under linux and windows. Mol. Ecol. Resour. 10, 564–567. 10.1111/j.1755-0998.2010.02847.x 21565059

[B19] Fang H. Liu H. Ma R. Liu Y. Li J. Yu X. (2020). Genome-wide assessment of population structure and genetic diversity of Chinese Lou onion using specific length amplified fragment (SLAF) sequencing. PLoS One 15, e0231753. 10.1371/journal.pone.0231753 32369481 PMC7199963

[B20] Gao Y. Wang D.-j. Wang K. Cong P.-h. Li L.-w. Piao J.-c. (2021). Analysis of genetic diversity and structure across a wide range of germplasm reveals genetic relationships among seventeen species of *Malus* Mill. native to China. J. Integr. Agric. 20, 3186–3198. 10.1016/s2095-3119(20)63421-9

[B21] Ge D. Wen Z. Xia L. Zhang Z. Erbajeva M. Huang C. (2013). Evolutionary history of lagomorphs in response to global environmental change. PLoS One 8, e59668. 10.1371/journal.pone.0059668 23573205 PMC3616043

[B22] Giannoulis T. Plageras D. Stamatis C. Chatzivagia E. Tsipourlianos A. Birtsas P. (2019). Islands and hybrid zones: Combining the knowledge from "natural laboratories" to explain phylogeographic patterns of the European brBrownare. BMC Evol. Biol. 19, 17. 10.1186/s12862-019-1354-y 30630408 PMC6329171

[B23] Hague M. T. Routman E. J. (2016). Does population size affect genetic diversity? A test with sympatric lizard species. Heredity 116, 92–98. 10.1038/hdy.2015.76 26306730 PMC4675878

[B24] Kardos M. (2021). Conservation genetics. Curr. Biol. 31, R1185–R1190. 10.1016/j.cub.2021.08.047 34637729

[B25] Kimura M. (1983). The neutral theory of molecular evolution. Cambridge University Press.

[B26] Kozlov A. M. Diego D. Tomáš F. Benoit M. Alexandros S. (2019). RAxML-NG: A fast, scalable, and user-friendly tool for maximum likelihood phylogenetic inference. Bioinformatics 35, 4453–4455. 10.1093/bioinformatics/btz305 31070718 PMC6821337

[B27] Kumar S. Stecher G. Li M. Knyaz C. Tamura K. (2018). Mega X: Molecular evolutionary genetics analysis across computing platforms. Mol. Biol. Evol. 35, 1547–1549. 10.1093/molbev/msy096 29722887 PMC5967553

[B28] Leigh J. W. Bryant D. (2015). PopART: Full-Feature software for haplotype network construction. Methods Ecol. Evol. 6, 1110–1116. 10.1111/2041-210x.12410

[B29] Li H. Durbin R. (2010). Fast and accurate long-read alignment with Burrows-Wheeler transform. Bioinformatics 26, 589–595. 10.1093/bioinformatics/btp698 20080505 PMC2828108

[B30] Li J. Wang K. Li Y. Sun G. Chu C. Li L. (2006). Geomorphological features, crustal composition and geological evolution of the Tianshan Mountains. Geol. Bull. China 25, 895–909. 10.3969/j.issn.1671-2552.2006.08.001 (in Chinese)

[B31] Li H. Handsaker B. Wysoker A. Fennell T. Ruan J. Homer N. (2009a). The sequence alignment/map format and SAMtools. Bioinformatics 25, 2078–2079. 10.1093/bioinformatics/btp352 19505943 PMC2723002

[B32] Li R. Yu C. Li Y. Lam T. W. Yiu S. M. Kristiansen K. (2009b). SOAP2: An improved ultrafast tool for short read alignment. Bioinformatics 25, 1966–1967. 10.1093/bioinformatics/btp336 19497933

[B33] Li Z. Liu Z. Wang M. Qian Z. Zhao P. Zhu Z. (2014). A review on studies of speciation in the presence of gene flow: Evolution of reproductive isolation. Biodivers. Sci. 22, 88–96. 10.3724/sp.j.1003.2014.13143 (in Chinese)

[B34] Li Z. Wei S. Li H. Wu K. Cai Z. Li D. (2017). Genome-wide genetic structure and differentially selected regions among Landrace, Erhualian, and Meishan pigs using specific-locus amplified fragment sequencing. Sci. Rep. 7, 10063. 10.1038/s41598-017-09969-6 28855565 PMC5577042

[B35] Liang Y. He D. Jia Y. Sun H. Chen Y. (2017). Phylogeographic studies of schizothoracine fishes on the central Qinghai-Tibet Plateau reveal the highest known glacial microrefugia. Sci. Rep. 7, 10983. 10.1038/s41598-017-11198-w 28887534 PMC5591315

[B36] Lindblad-Toh K. Garber M. Zuk O. Lin M. F. Parker B. J. Washietl S. (2011). A high-resolution map of human evolutionary constraint using 29 mammals. Nature 478, 476–482. 10.1038/nature10530 21993624 PMC3207357

[B37] Liu J. Yu L. Arnold M. L. Wu C. H. Wu S. F. Lu X. (2011). Reticulate evolution: Frequent introgressive hybridization among Chinese hares (genus *lepus*) revealed by analyses of multiple mitochondrial and nuclear DNA loci. BMC Evol. Biol. 11, 1–14. 10.1186/1471-2148-11-223 21794180 PMC3155923

[B38] Liu K. Muse S. V. (2005). PowerMarker: An integrated analysis environment for genetic marker analysis. Bioinformatics 21, 2128–2129. 10.1093/bioinformatics/bti282 15705655

[B39] McKenna A. Hanna M. Banks E. Sivachenko A. Cibulskis K. Kernytsky A. (2010). The genome analysis toolkit: A MapReduce framework for analyzing next-generation DNA sequencing data. Genome Res. 20, 1297–1303. 10.1101/gr.107524.110 20644199 PMC2928508

[B40] Meiri M. Lister A. Kosintsev P. Zazula G. Barnes I. (2020). Population dynamics and range shifts of moose (*Alces alces*) during the Late Quaternary. J. Biogeogr. 47, 2223–2234. 10.1111/jbi.13935

[B41] Minoudi S. Papapetridis L. Karaiskou N. Chatzinikos E. Triantaphyllidis C. Abatzopoulos T. J. (2018). Genetic analyses of Brown hare (*Lepus europaeus*) support limited migration and translocation of Greek populations. PLoS One 13, e0206327. 10.1371/journal.pone.0206327 30379887 PMC6209229

[B42] Nadachowska K. Babik W. (2009). Divergence in the face of gene flow: The case of two newts (amphibia: Salamandridae). Mol. Biol. Evol. 26, 829–841. 10.1093/molbev/msp004 19136451

[B43] Nguyen L. T. Schmidt H. A. von Haeseler A. Minh B. Q. (2015). IQ-TREE: A fast and effective stochastic algorithm for estimating maximum-likelihood phylogenies. Mol. Biol. Evol. 32, 268–274. 10.1093/molbev/msu300 25371430 PMC4271533

[B44] Nosil P. (2008). Speciation with gene flow could be common. Mol. Ecol. 17, 2103–2106. 10.1111/j.1365-294X.2008.03715.x 18410295

[B45] Pickrell J. K. Pritchard J. K. (2012). Inference of population splits and mixtures from genome-wide allele frequency data. PLoS Genet. 8, e1002967. 10.1371/journal.pgen.1002967 23166502 PMC3499260

[B46] Pierpaoli M. Riga F. Trocchi V. Randi E. (2003). Hare populations in Europe: Intra and interspecific analysis of mtDNA variation. C. R. Biol. 326, 80–84. 10.1016/s1631-0691(03)00042-8 14558454

[B47] Pironon S. Papuga G. Villellas J. Angert A. L. Garcia M. B. Thompson J. D. (2017). Geographic variation in genetic and demographic performance: New insights from an old biogeographical paradigm. Biol. Rev. Camb. Philos. Soc. 92, 1877–1909. 10.1111/brv.12313 27891813

[B48] Purcell S. Neale B. Todd-Brown K. Thomas L. Ferreira M. A. Bender D. (2007). Plink: A tool set for whole-genome association and population-based linkage analyses. Am. J. Hum. Genet. 81, 559–575. 10.1086/519795 17701901 PMC1950838

[B49] Qin M. Li C. Li Z. Chen W. Zeng Y. (2019). Genetic diversities and differentially selected regions between shandong indigenous pig breeds and western pig breeds. Front. Genet. 10, 1351–1361. 10.3389/fgene.2019.01351 32038711 PMC6987402

[B50] Rambaut A. Drummond A. J. Xie D. Baele G. Suchard M. A. (2018). Posterior summarization in bayesian phylogenetics using tracer 1.7. Syst. Biol. 67, 901–904. 10.1093/sysbio/syy032 29718447 PMC6101584

[B51] Ren L. Li X. Kang L. Brunson K. Liu H. Dong W. (2017). Human paleodiet and animal utilization strategies during the Bronze Age in northwest Yunnan Province, southwest China. PLoS One 12, e0177867. 10.1371/journal.pone.0177867 28531221 PMC5439680

[B52] Ren A. Du K. Jia X. Yang R. Wang J. Chen S. Y. (2019). Genetic diversity and population structure of four Chinese rabbit breeds. PLoS One 14, e0222503. 10.1371/journal.pone.0222503 31525233 PMC6746397

[B53] Ronquist F. Teslenko M. van der Mark P. Ayres D. L. Darling A. Hohna S. (2012). MrBayes 3.2: Efficient bayesian phylogenetic inference and model choice across a large model space. Syst. Biol. 61, 539–542. 10.1093/sysbio/sys029 22357727 PMC3329765

[B54] Rozas J. Ferrer-Mata A. Sanchez-DelBarrio J. C. Guirao-Rico S. Librado P. Ramos-Onsins S. E. (2017). DnaSP 6: DNA sequence polymorphism analysis of large data sets. Mol. Biol. Evol. 34, 3299–3302. 10.1093/molbev/msx248 29029172

[B55] Sarabia C. vonHoldt B. Larrasoana J. C. Urios V. Leonard J. A. (2021). Pleistocene climate fluctuations drove demographic history of African golden wolves (*Canis lupaster*). Mol. Ecol. 30, 6101–6120. 10.1111/mec.15784 33372365

[B56] Shan W. Liu J. Yu L. Robert W. M. Mahmut H. Zhang Y. (2011). Genetic consequences of postglacial colonization by the endemic Yarkand hare (*Lepus yarkandensis*) of the arid Tarim Basin. Chin. Sci. Bull. 56, 1370–1382. 10.1007/s11434-011-4460-9

[B57] Shan W. Dai H. Zhang Y. (2020a). Classification and genetic diversity of three hare species in Xinjiang based on mitochondrial DNA. Acta Veterinaria Zootechnica Sinica 51, 80–89. 10.11843/j.issn.0366-6964.2020.10.008 (in Chinese)

[B58] Shan W. Tursun M. Zhou S. Zhang Y. Dai H. (2020b). The complete mitochondrial genome sequence of *Lepus tolai* in Xinjiang. Mitochondrial DNA Part B 5, 1336–1337. 10.1080/23802359.2020.1735267

[B59] Smith A. T. Johnston C. H. Alves P. C. Hacklander K. (2018). Lagomorphs: Pikas, rabbits, and hares of the world. Baltimore: Johns Hopkins University Press.

[B60] Smith A. T. Xie Y. (2008). A guide to the mammals of China (in Chinese).

[B61] Suchard M. A. Lemey P. Baele G. Ayres D. L. Drummond A. J. Rambaut A. (2018). Bayesian phylogenetic and phylodynamic data integration using BEAST 1.10. Virus Evol. 4, vey016. 10.1093/ve/vey016 29942656 PMC6007674

[B62] Sun X. Liu D. Zhang X. Li W. Liu H. Hong W. (2013). SLAF-Seq: An efficient method of large-scale de novo SNP discovery and genotyping using high-throughput sequencing. PLoS One 8, e58700. 10.1371/journal.pone.0058700 23527008 PMC3602454

[B63] Tan B. Wang H. Wang X. Yi S. Zhou J. Ma C. (2022). The study of early human settlement preference and settlement prediction in Xinjiang, China. Sci. Rep. 12, 5072. 10.1038/s41598-022-09033-y 35332226 PMC8948180

[B64] Terhorst J. Kamm J. A. Song Y. S. (2017). Robust and scalable inference of population history from hundreds of unphased whole genomes. Nat. Genet. 49, 303–309. 10.1038/ng.3748 28024154 PMC5470542

[B65] Wang J. Yang G. (2012). Complete mitogenome of cape hare *Lepus capensis* ( Lagomorpha: Leporidae) and its phylogenetic considerations. Acta Theriol. Sin. 32, 1–11. 10.16829/j.slxb.2012.01.001

[B66] Wang W. Zhang T. Wang J. Zhang G. Wang Y. Zhang Y. (2016). Genome-wide association study of 8 carcass traits in Jinghai Yellow chickens using specific-locus amplified fragment sequencing technology. Poult. Sci. 95, 500–506. 10.3382/ps/pev266 26614681 PMC4957485

[B67] Wu C. Wu J. Bunch T. D. Li Q. Wang Y. Zhang Y. P. (2005). Molecular phylogenetics and biogeography of *Lepus* in Eastern Asia based on mitochondrial DNA sequences. Mol. Phylogenet. Evol. 37, 45–61. 10.1016/j.ympev.2005.05.006 15990340

[B68] Wu Y. Xia L. Zhang Q. Yang Q. Meng X. (2011). Bidirectional introgressive hybridization between *Lepus capensis* and *Lepus yarkandensis* . Mol. Phylogenet. Evol. 59, 545–555. 10.1016/j.ympev.2011.03.027 21463697

[B69] Xie H. X. Liang X. X. Chen Z. Q. Li W. M. Mi C. R. Li M. (2022). Ancient demographics determine the effectiveness of genetic purging in endangered Lizards. Mol. Biol. Evol. 39, msab359. 10.1093/molbev/msab359 34919713 PMC8788223

[B70] Yamada T. Maki M. (2012). Impact of geographical isolation on genetic differentiation in insular and mainland populations of *Weigela coraeensis* (Caprifoliaceae) on Honshu and the Izu Islands. J. Biogeogr. 39, 901–917. 10.1111/j.1365-2699.2011.02634.x

[B71] Yuan J. Ye Z. Bu W. (2019). Phylogeography of widespread species in Eurasia: Current progress and future prospects. Sci. Sin. -Vitae. 49, 1155–1164. 10.1360/ssv-2019-0163 (in Chinese)

[B72] Zhang D. Zhang Z. (2005). Single nucleotide polymorphisms (SNPs) discovery and linkage disequilib-rium (LD) in forest trees. For. Stud. China 7, 1–14. 10.1007/s11632-005-0024-x

[B73] Zhang Y. Wang L. Xin H. Li D. Ma C. Ding X. (2013). Construction of a high-density genetic map for sesame based on large scale marker development by specific length amplified fragment (SLAF) sequencing. BMC Plant Biol. 13, 141–152. 10.1186/1471-2229-13-141 24060091 PMC3852768

[B74] Zhang D. Gao F. Li W. Jakovlić I. Zou H. Zhang J. (2018). PhyloSuite: An integrated and scalable desktop platform for streamlined molecular sequence data management and evolutionary phylogenetics studies. Mol. Ecol. Resour. 20, 348–355. 10.1111/1755-0998.13096 31599058

[B75] Zhang J. Sun B. Li C. Chen W. Jiang L. Lv S. (2020a). Molecular diversity and genetic structure of wild rice accessions (*Oryza rufipogon* Griff.) in Guangdong Province, China, as revealed by SNP markers. Genet. Resour. Crop Evol. 68, 969–978. 10.1007/s10722-020-01038-8

[B76] Zhang Y. Zeng W. Xu P. Alemujiang G. Shan W. (2020b). The screening of DNA barcode for hares in Xinjiang. Acta Veterinaria Zootechnica Sinica 51, 270–278. 10.11843/j.issn.0366-6964.2020.02.008 (in Chinese)

[B77] Zhao S. Zheng P. Dong S. Zhan X. Wu Q. Guo X. (2013). Whole-genome sequencing of giant pandas provides insights into demographic history and local adaptation. Nat. Genet. 45, 67–71. 10.1038/ng.2494 23242367

