# Corrigendum: Population genetics analysis of Tolai hares (*Lepus tolai*) in Xinjiang, China using genome-wide SNPs from SLAF-seq and mitochondrial markers

**DOI:** 10.3389/fgene.2023.1179564

**Published:** 2023-03-20

**Authors:** Miregul Mamat, Wenjuan Shan, Pengcheng Dong, Shiyu Zhou, Peng Liu, Yang Meng, Wenyue Nie, Peichen Teng, Yucong Zhang

**Affiliations:** Xinjiang Key Laboratory of Biological Resources and Genetic Engineering, College of Life Science and Technology, Xinjiang University, Urumqi, China

**Keywords:** *Lepus tolai*, SLAF-seq, mtDNA, genetic diversity, genetic structure

In the published article, there was an error in Figure 3C as published. A “B” is missing from the note representing the BRJ population.

**FIGURE 3:**
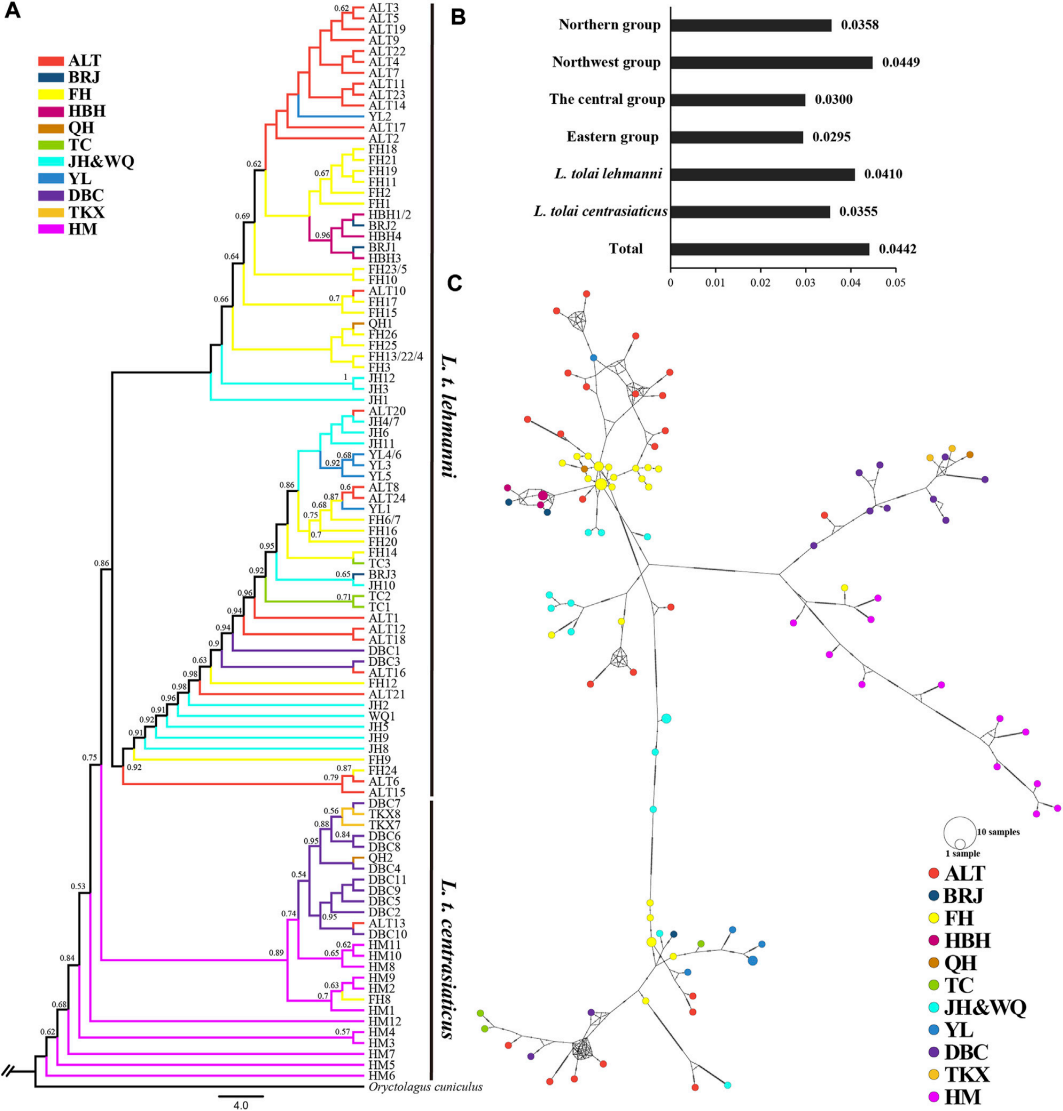
Genetic diversity and population structure of Tolai hares in Xinjiang based on mtDNA. **(A)**. Phylogenctic tree constructed using ML method. **(B)**. Nucleotide diversity (π). **(C)**. Median-joining network of Tolai hare haplotypes. The hatch marks on the line indicates the mutation numbers.

The corrected Figure 3C and its caption “Genetic diversity and population structure of Tolai hares in Xinjiang based on mtDNA. **(A)**. Phylogenctic tree constructed using ML method. **(B)**. Nucleotide diversity (π). **(C)**. Median-joining network of Tolai hare haplotypes. The hatch marks on the line indicates the mutation numbers.” appear below.

In the published article, there was an error. The sentence was misstated, one word was miswritten, *ND4* was written as *CYTB*.

A correction has been made to **Materials and methods**, Mitochondrial DNA sequencing, First Paragraph. This sentence previously stated:

“PCR primers for *CYTB* were 5′-GCA​AAG​AAT​CAT​TAC​TAC​GCA​AA-3′ (F) and 5′-TTG​CGA​CGA​TTA​CTA​AGG​CTA-3′ (R) (Zhang Y. et al., 2020b)”

The corrected sentence appears below:

“PCR primers for *ND4* were 5′-GCA​AAG​AAT​CAT​TAC​TAC​GCA​AA-3′ (F) and 5′-TTG​CGA​CGA​TTA​CTA​AGG​CTA-3′ (R) (Zhang Y. et al., 2020b)”

The authors apologize for this error and state that this does not change the scientific conclusions of the article in any way. The original article has been updated.

