# Supplementary material for: Population genetics analysis of Tolai hares (Lepus tolai) in Xinjiang, China using genome-wide SNPs from SLAF-seq and mitochondrial markers
Source: Front Genet. 2023 Mar 20;13:1018632. doi: 10.3389/fgene.2022.1018632 (PMC10064446; doi:10.3389/fgene.2022.1018632)
Supplement: Supplementary file 1 [file DataSheet1.ZIP › 2. Additional file 2:Table S2.docx]

**Additional File2: Table S2**

Accession number of SNPs and mtDNA data for the Xinjiang Tolai hare

| **Data type** | **Accession number** | | | |
| --- | --- | --- | --- | --- |
| SLAF-seq | PRJNA850843 | | | |
| mtDNA | *COI* | *ND4* | *CYTB* | D-LOOP |
| ALT1 | OP585917 | OP302843 | OP302949 | OP303055 |
| ALT2 | OP594653 | OP302844 | OP302950 | OP303056 |
| ALT3 | OP594654 | OP302845 | OP302951 | OP303057 |
| ALT4 | OP600582 | OP302846 | OP302952 | OP303058 |
| ALT5 | OP594655 | OP302847 | OP302953 | OP303059 |
| ALT6 | OP600583 | OP302848 | OP302954 | OP303060 |
| ALT7 | OP594656 | OP302849 | OP302955 | OP303061 |
| ALT8 | OP600584 | OP302850 | OP302956 | OP303062 |
| ALT9 | OP600585 | OP302851 | OP302957 | OP303063 |
| ALT10 | OP594657 | OP302852 | OP302958 | OP303064 |
| ALT11 | OP594658 | OP302853 | OP302959 | OP303065 |
| ALT12 | OP594659 | OP302854 | OP302960 | OP303066 |
| ALT13 | OP594660 | OP302855 | OP302961 | OP303067 |
| ALT14 | OP594661 | OP302856 | OP302962 | OP303068 |
| ALT15 | OP594662 | OP302857 | OP302963 | OP303069 |
| ALT16 | OP594663 | OP302858 | OP302964 | OP303070 |
| ALT17 | OP594664 | OP302859 | OP302965 | OP303071 |
| ALT18 | OP594665 | OP302860 | OP302966 | OP303072 |
| ALT19 | OP594666 | OP302861 | OP302967 | OP303073 |
| ALT20 | OP600586 | OP302862 | OP302968 | OP303074 |
| ALT21 | OP600587 | OP302863 | OP302969 | OP303075 |
| ALT22 | OP600588 | OP302864 | OP302970 | OP303076 |
| ALT23 | OP600589 | OP302865 | OP302971 | OP303077 |
| ALT24 | OP594667 | OP302866 | OP302972 | OP303078 |
| BRJ1 | OP600590 | OP302867 | OP302973 | OP303079 |
| BRJ2 | OP600591 | OP302868 | OP302974 | OP303080 |
| BRJ3 | OP600592 | OP302869 | OP302975 | OP303081 |
| FH1 | OP600593 | OP302870 | OP302976 | OP303082 |
| FH2 | OP594668 | OP302871 | OP302977 | OP303083 |
| FH3 | OP594669 | OP302872 | OP302978 | OP303084 |
| FH4 | OP594670 | OP302873 | OP302979 | OP303085 |
| FH5 | OP594671 | OP302874 | OP302980 | OP303086 |
| FH6 | OP594672 | OP302875 | OP302981 | OP303087 |
| FH7 | OP594673 | OP302876 | OP302982 | OP303088 |
| FH8 | OP594674 | OP302877 | OP302983 | OP303089 |
| FH9 | OP594675 | OP302878 | OP302984 | OP303090 |
| FH10 | OP594676 | OP302879 | OP302985 | OP303091 |
| FH11 | OP594677 | OP302880 | OP302986 | OP303092 |
| FH12 | OP594678 | OP302881 | OP302987 | OP303093 |
| FH13 | OP594679 | OP302882 | OP302988 | OP303094 |
| FH14 | OP594680 | OP302883 | OP302989 | OP303095 |
| FH15 | OP600594 | OP302884 | OP302990 | OP303096 |
| FH16 | OP600595 | OP302885 | OP302991 | OP303097 |
| FH17 | OP600596 | OP302886 | OP302992 | OP303098 |
| FH18 | OP594681 | OP302887 | OP302993 | OP303099 |
| FH19 | OP600597 | OP302888 | OP302994 | OP303100 |
| FH20 | OP600598 | OP302889 | OP302995 | OP303101 |
| FH21 | OP594682 | OP302890 | OP302996 | OP303102 |
| FH22 | OP594683 | OP302891 | OP302997 | OP303103 |
| FH23 | OP594684 | OP302892 | OP302998 | OP303104 |
| FH24 | OP594685 | OP302893 | OP302999 | OP303105 |
| FH25 | OP600599 | OP302894 | OP303000 | OP303106 |
| FH26 | OP600600 | OP302895 | OP303001 | OP303107 |
| HBH1 | OP594686 | OP302896 | OP303002 | OP303108 |
| HBH2 | OP594687 | OP302897 | OP303003 | OP303109 |
| HBH3 | OP600601 | OP302898 | OP303004 | OP303110 |
| HBH4 | OP600602 | OP302899 | OP303005 | OP303111 |
| QH1 | OP600612 | OP302900 | OP303006 | OP303112 |
| QH2 | OP594688 | OP302901 | OP303007 | OP303113 |
| TC1 | OP594689 | OP302902 | OP303008 | OP303114 |
| TC2 | OP600613 | OP302903 | OP303009 | OP303115 |
| TC3 | OP600614 | OP302904 | OP303010 | OP303116 |
| JH1 | OP594690 | OP302905 | OP303011 | OP303117 |
| JH2 | OP594691 | OP302906 | OP303012 | OP303118 |
| JH3 | OP594692 | OP302907 | OP303013 | OP303119 |
| JH4 | OP594693 | OP302908 | OP303014 | OP303120 |
| JH5 | OP594694 | OP302909 | OP303015 | OP303121 |
| JH6 | OP594695 | OP302910 | OP303016 | OP303122 |
| JH7 | OP594696 | OP302911 | OP303017 | OP303123 |
| JH8 | OP594697 | OP302912 | OP303018 | OP303124 |
| JH9 | OP594698 | OP302913 | OP303019 | OP303125 |
| JH10 | OP594699 | OP302914 | OP303020 | OP303126 |
| JH11 | OP600611 | OP302915 | OP303021 | OP303127 |
| JH12 | OP594700 | OP302916 | OP303022 | OP303128 |
| WQ1 | OP600615 | OP302917 | OP303023 | OP303129 |
| YL1 | OP594701 | OP302918 | OP303024 | OP303130 |
| YL2 | OP600616 | OP302919 | OP303025 | OP303131 |
| YL3 | OP600617 | OP302920 | OP303026 | OP303132 |
| YL4 | OP600618 | OP302921 | OP303027 | OP303133 |
| YL5 | OP600619 | OP302922 | OP303028 | OP303134 |
| YL6 | OP600620 | OP302923 | OP303029 | OP303135 |
| DBC1 | OP594702 | OP302924 | OP303030 | OP303136 |
| DBC2 | OP594703 | OP302925 | OP303031 | OP303137 |
| DBC3 | OP594704 | OP302926 | OP303032 | OP303138 |
| DBC4 | OP594705 | OP302927 | OP303033 | OP303139 |
| DBC5 | OP594706 | OP302928 | OP303034 | OP303140 |
| DBC6 | OP594707 | OP302929 | OP303035 | OP303141 |
| DBC7 | OP594708 | OP302930 | OP303036 | OP303142 |
| DBC8 | OP594709 | OP302931 | OP303037 | OP303143 |
| DBC9 | OP594710 | OP302932 | OP303038 | OP303144 |
| DBC10 | OP594711 | OP302933 | OP303039 | OP303145 |
| DBC11 | OP594712 | OP302934 | OP303040 | OP303146 |
| TKX7 | OP594713 | OP302935 | OP303041 | OP303147 |
| TKX8 | OP594714 | OP302936 | OP303042 | OP303148 |
| HM1 | OP594715 | OP302937 | OP303043 | OP303149 |
| HM2 | OP600603 | OP302938 | OP303044 | OP303150 |
| HM3 | OP594716 | OP302939 | OP303045 | OP303151 |
| HM4 | OP600604 | OP302940 | OP303046 | OP303152 |
| HM5 | OP600605 | OP302941 | OP303047 | OP303153 |
| HM6 | OP594717 | OP302942 | OP303048 | OP303154 |
| HM7 | OP600606 | OP302943 | OP303049 | OP303155 |
| HM8 | OP600607 | OP302944 | OP303050 | OP303156 |
| HM9 | OP600608 | OP302945 | OP303051 | OP303157 |
| HM10 | OP600609 | OP302946 | OP303052 | OP303158 |
| HM11 | OP600610 | OP302947 | OP303053 | OP303159 |
| HM12 | OP594718 | OP302948 | OP303054 | OP303160 |
