# Supplementary figures and images for: Population genetics analysis of Tolai hares (Lepus tolai) in Xinjiang, China using genome-wide SNPs from SLAF-seq and mitochondrial markers
Source: Front Genet. 2023 Mar 20;13:1018632. doi: 10.3389/fgene.2022.1018632 (PMC10064446; doi:10.3389/fgene.2022.1018632)

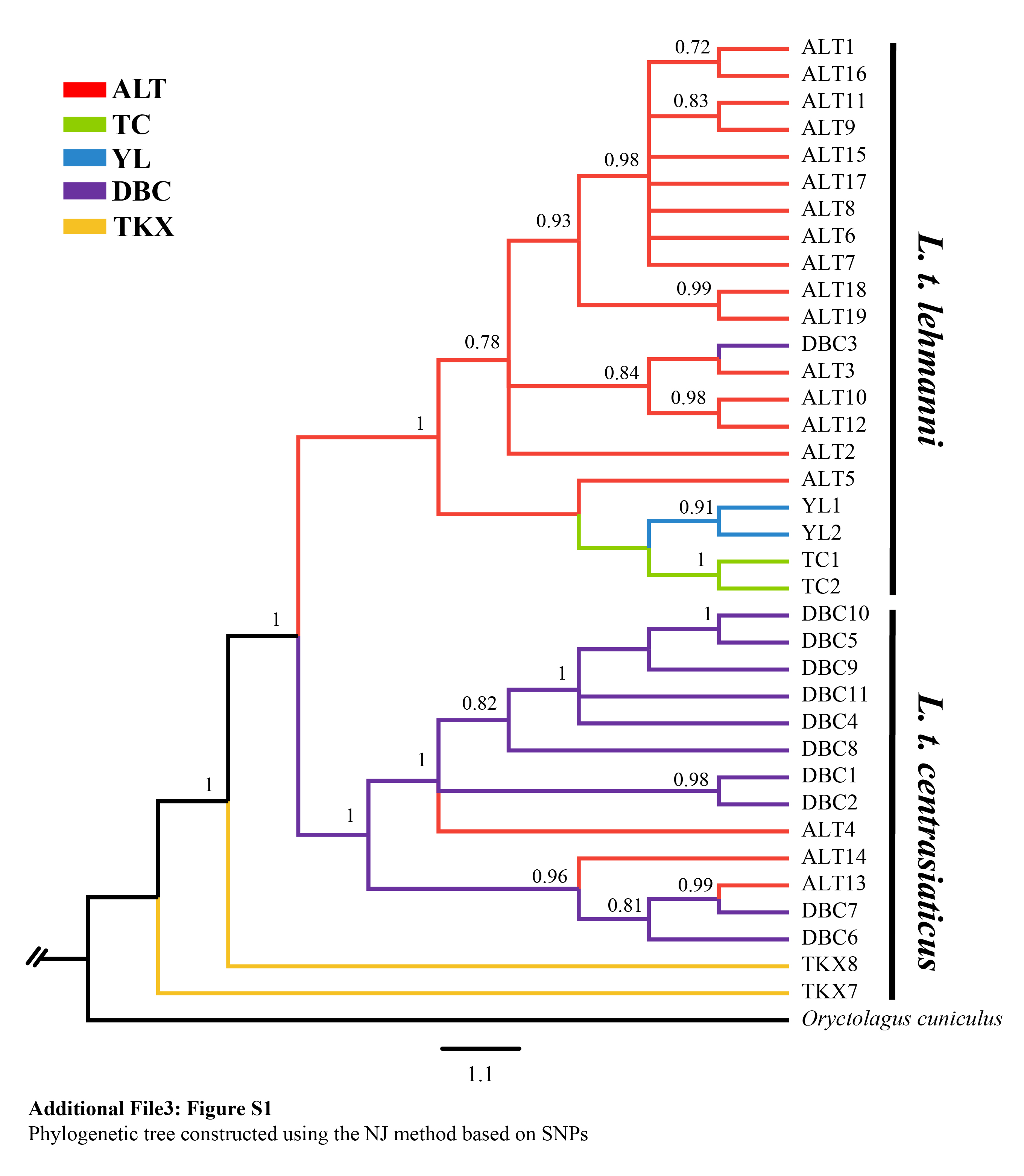

Supplement: Supplementary file 1 [file DataSheet1.ZIP › 3. Additional File3 Figure S1 .jpg]

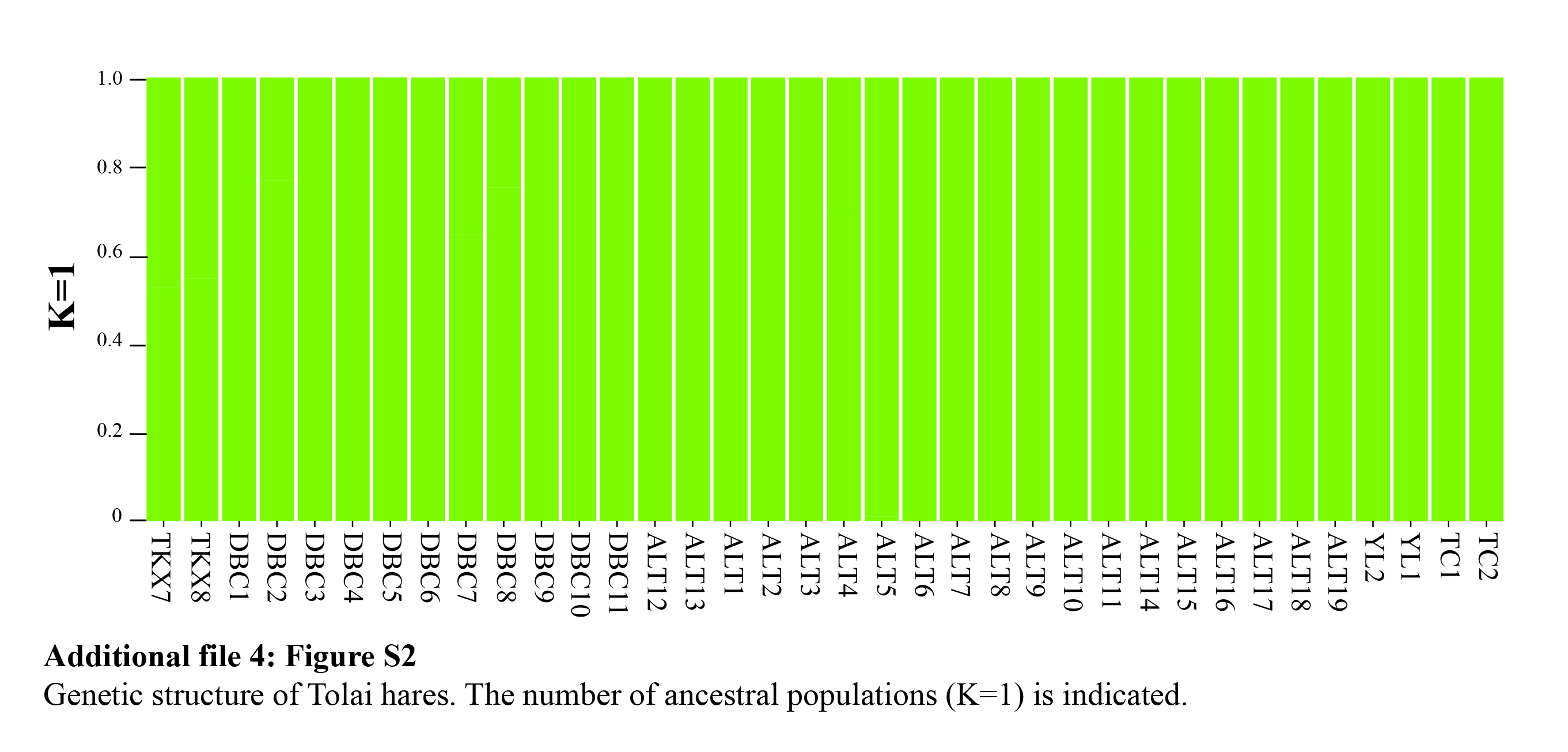

Supplement: Supplementary file 1 [file DataSheet1.ZIP › 4. Additional file 4 Figure S2.jpg]

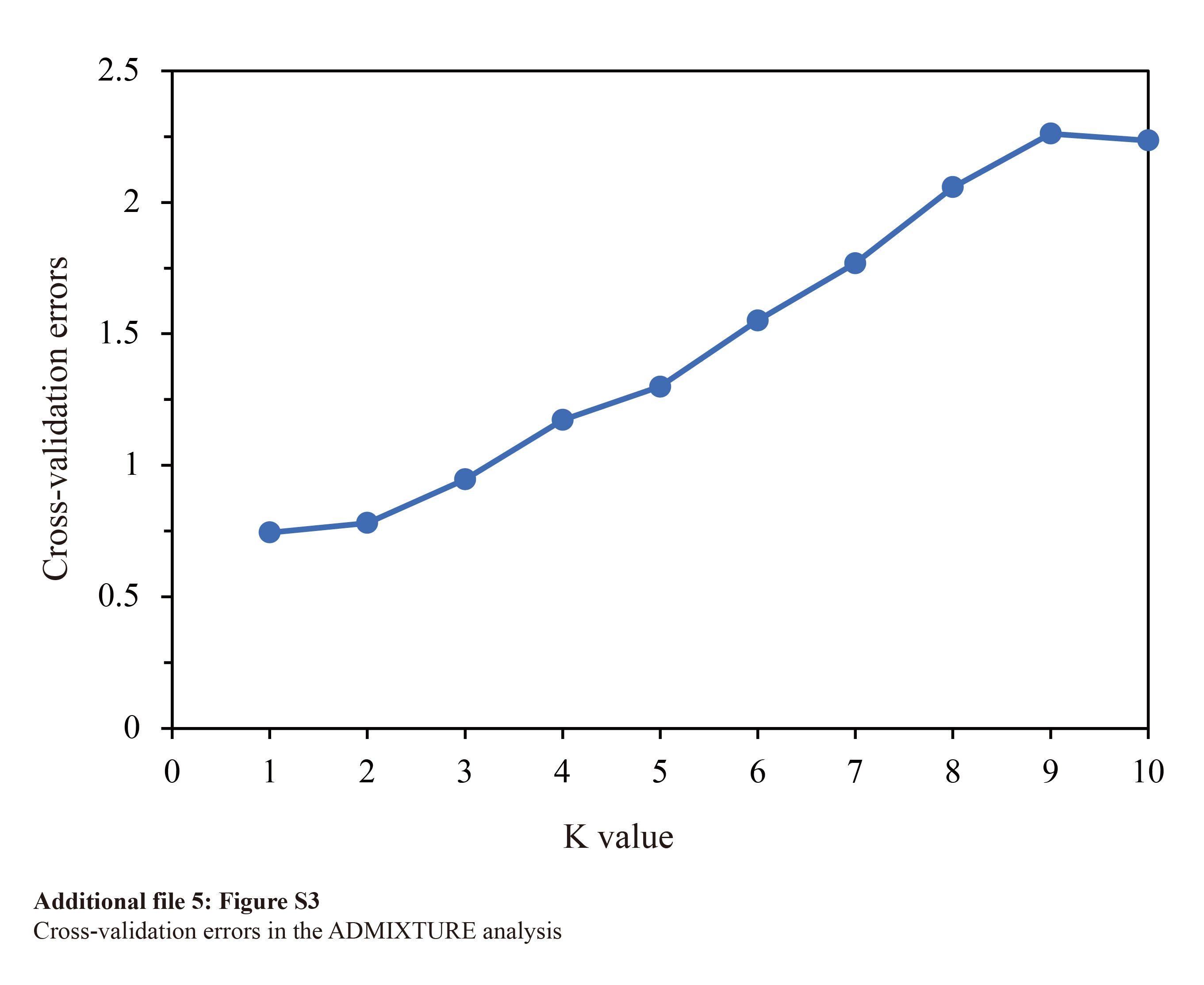

Supplement: Supplementary file 1 [file DataSheet1.ZIP › 5. Additional file 5 Figure S3 .jpg]

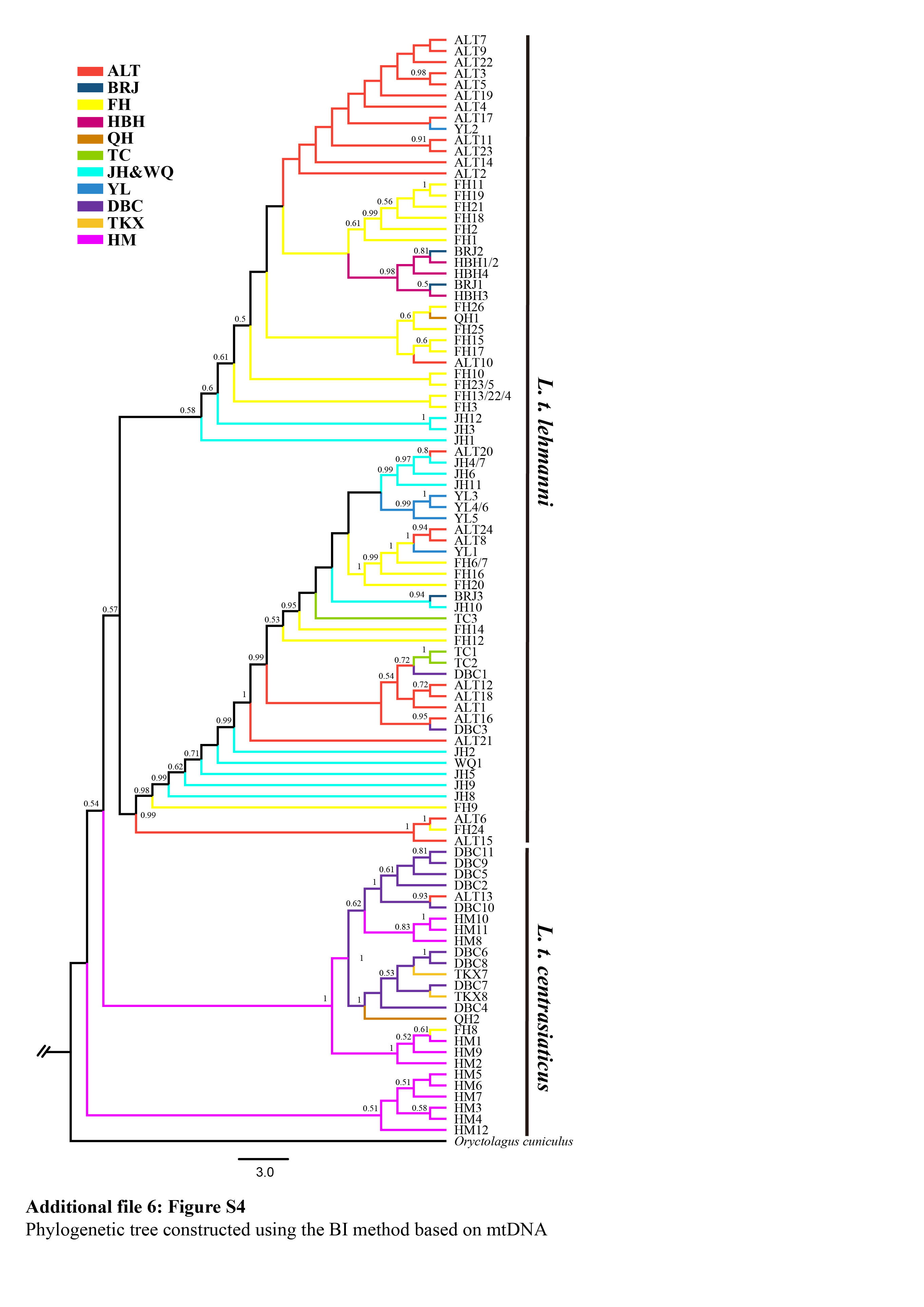

Supplement: Supplementary file 1 [file DataSheet1.ZIP › 6. Additional file 6 Figure S4 .jpg]

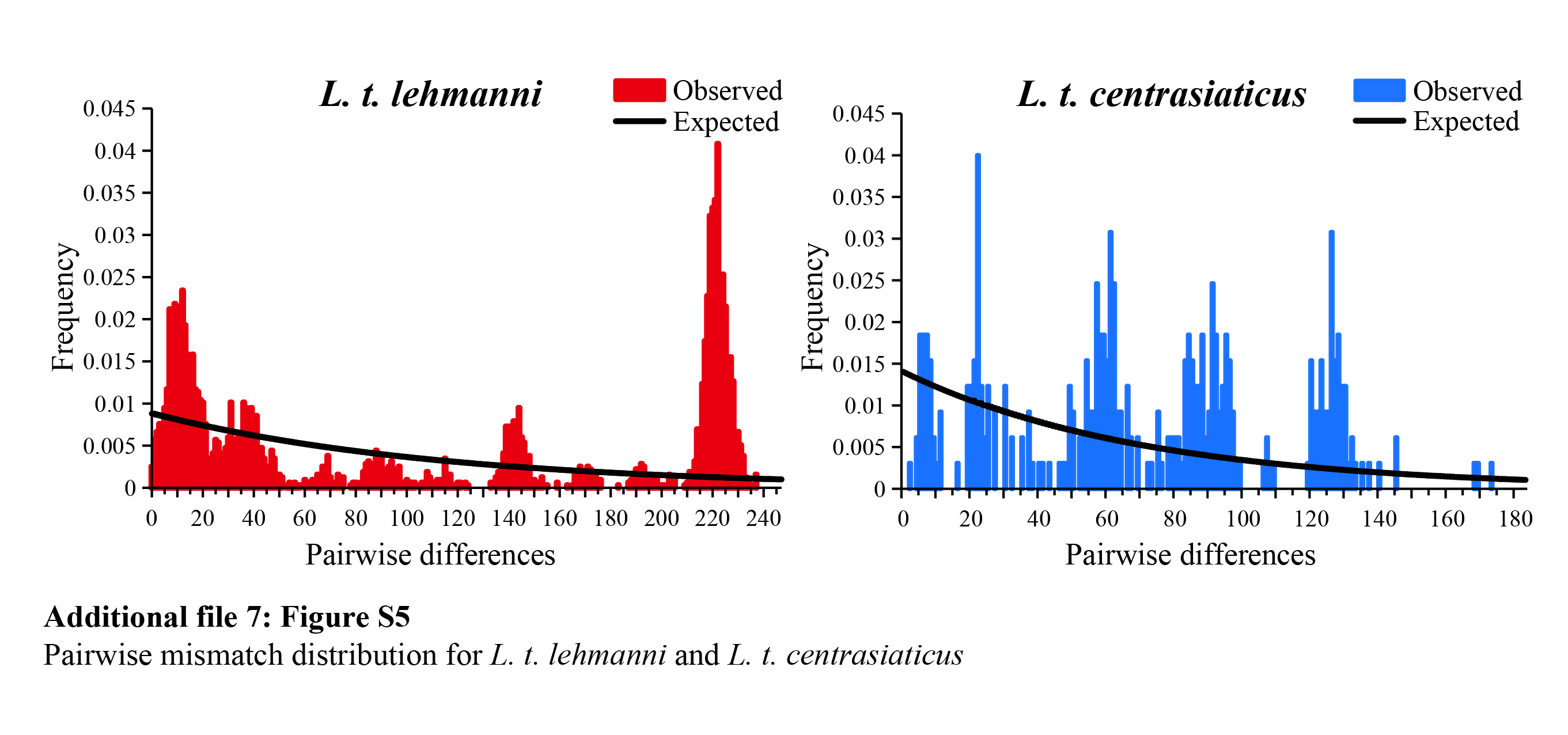

Supplement: Supplementary file 1 [file DataSheet1.ZIP › 7. Additional file 7 Figure S5 .jpg]
